# Cognitive pediatric tele-assessment: a scoping review

**DOI:** 10.3389/fpsyg.2023.1288021

**Published:** 2023-12-15

**Authors:** Nestor Viñas-Guasch, Phoebe Si Qi Chia, Michelle Li-Mei Yap, Chiao-Yi Wu, S. H. Annabel Chen

**Affiliations:** ^1^Psychology, School of Social Sciences, Nanyang Technological University, Singapore, Singapore; ^2^Department of Rehabilitation Sciences, The Hong Kong Polytechnic University, Hong Kong, Hong Kong SAR, China; ^3^Centre for Research in Child Development, National Institute of Education, Nanyang Technological University, Singapore, Singapore; ^4^Centre for Research and Development in Learning (CRADLE), Nanyang Technological University, Singapore, Singapore; ^5^Lee Kong Chian School of Medicine (LKCMedicine), Nanyang Technological University, Singapore, Singapore

**Keywords:** cognitive tele-assessment, remote, cognition, pediatric, children

## Abstract

Cognitive tele-assessment (CTA) adoption has increased considerably recently, in parallel with the maturation of the digital technologies that enable it, and the push to move assessment to the online format during the COVID-19 pandemic in 2019. This mode of assessment stems from remote assessment applications that originated in general tele-medicine, where it was typically used for patient screening as part of an intervention. The development of remote tele-medicine was later adapted for CTA in adult populations in tele-neuropsychiatry and tele-psychology and is increasingly applied in experimental research in cognitive science research with adult and pediatric populations, and for remote academic assessment. Compared to in-person assessment, CTA offers advantages such as decreasing time and logistic costs and facilitating the assessment of remote or special needs populations. However, given the novelty of CTA, its technical, methodological, and ethical issues remain poorly understood, especially in cases where methods for assessment of adults are used in pediatric populations. In the current paper, we provide a scoping review on the evolution of remote tele-assessment from the years 2000 to 2021, to identify its main themes, methodologies, and applications, and then focus on the issues of assessment in pediatric populations. Finally, we present recommendations on how to address the challenges previously mentioned.

## 1 Introduction

One of the recent trends in experimental research in cognitive science is a progressive shift from presential testing to cognitive tele-assessment (CTA). CTA has been defined as the assessment of cognitive functions that is conducted through the online medium rather than presentially (Krach et al., 2020; Ruffini et al., 2022). In this context, cognitive functions are defined as mental processes responsible for perception, storage, retrieval and manipulation of information from the environment, such as perception, memory, attention, learning, decision making and language abilities (Kiely et al., 2014).

While CTA is a new phenomenon in cognitive science research, many of its current methods can be traced back to the practice of tele-medicine that surged in the 1950’s and 1960’s, when the Nebraska Psychiatric Institute implemented a closed-circuit TV system between two hospitals so that clinicians in one hospital could interview patients located in the other hospital (Wurm et al., 2008). A series of similar projects, additionally incorporating satellite technology were implemented from the 1970’s through the 1990’s, where online assessment tests were included as part of broader therapeutic interventions (Hodge et al., 2019). However, the high cost of the technical equipment rendered these projects unfeasible in many cases (Wurm et al., 2008).

The recent increase in CTA adoption can be explained by both the maturation of technology that makes CTA possible, and more recently, the introduction of restrictions in face-to-face testing at the start of the COVID-19 pandemic. Along these lines, the present study addresses the timeframe spanning from years 2000 to 2021, divided into two periods. The first period (years 2000–2019) encapsulates the time when CTA adoption was mainly driven by technological advances such as the shift from dial-up internet (prevalent in the 1990’s) to much faster Digital Subscriber Line (DSL) and cable internet technologies, the development and expansion of optic fiber and cable network reach by Internet Service Provider companies, the decrease of the costs of high speed broadband and increased demand for fast internet connections such as streaming media services, and finally the introduction of mobile devices, typically cheaper and globally more accessible than traditional computers.

The second period of time (2019–2021) marks a transition influenced by the onset of the COVID-19 pandemic, and the sudden restrictions in face-to-face testing at the start of the pandemic, which catalyzed a shift in learning, teaching, and research from the presential to the online format (Lemay et al., 2021; Webb et al., 2021). And altered global needs, habits, and healthcare practices, including the delivery of cognitive assessments for children. In this context, the need to minimize in-person contact led to rapid adoption of teleassessment tools and platforms. The pandemic had a deep impact on people’s habits and needs in a wide range of domains. As a result of the need to minimize in person contact and exposure to avoid spread of infection, physical distancing measures and lockdowns were implemented, which had a profound effect on global needs, and altered peoples’ daily routines and ingrained habits. For instance, to compensate for the lack of face-to-face interaction, social media, instant messaging and video calls became primary forms of social interaction. In addition, working from home became the default mode of work, thus accelerating the adoption of videoconferencing and online collaborative tools. Finally, there was a surge in the provision of remote telehealth services, with an increase in virtual consultations and telemedicine, and access to online therapy and counseling services. In this context, the closure of schools and other educational institutions led to a shift toward online education and assessment, with focus on video conferencing, online platforms, and digital resources, and forcing educators and researchers to move assessment materials to the online medium. As this was our experience in our research on pediatric neuroimaging (MRI), the present review focuses on these two last domains, namely the cognitive assessment of pediatric populations both in educational and clinical settings.

It is widely acknowledged that CTA offers many benefits over presential testing. Specifically, CTA has the potential to increase accessibility and reach to a wider population, by reducing travel time, providing more flexible scheduling and reducing the cost of assessment, thus enabling access for people with transportation challenges or reduced mobility. In addition, CTA has the potential to reduce social stigma or performance anxiety, since assessment occurs at the participant’s home, which is a more familiar setting. In addition, CTA makes standardization easier, given that CTA is conducted online, and administration, scoring and data analysis can be standardized and shared across research groups if necessary.

Despite its advantages, CTA also has shortcomings compared to traditional presential assessment. For instance, the reliance on technology makes it more susceptible to technical problems (internet outages, software and hardware bugs and incompatibilities) and increases the risk of data and privacy breaches when transmitting personal information on the internet. Whereas improved technology and rigorous data handling practices can mitigate these problems, a more pervasive issue concerns equity in access to CTA services. Benefitting from the advantages of CTA requires access to the internet, suitable digital devices and home environment, but this is hampered by large disparities in populations with different socioeconomic or cultural backgrounds, or without the required technical proficiency to operate the devices, such as elderly or impoverished people, or people living in rural areas with inadequate internet coverage. Likewise, CTA is largely unavailable to people who are physically unable to use the devices, such as patients with motor or neurological disorders.

Another factor that limits CTA is the limited ‘hands-on’ interaction between researcher/clinician and the participant. This factor makes it difficult for the experimenter to control the assessment environment, such as potential distractions that the participant might be exposed to in the home setting. In addition, CTA is associated with lower interaction between experimenter and participant, which might lead to lower levels of rapport and decreased engagement and motivation to complete the task. Similarly, lack of in-person interaction severely limits CTA in tests that require physical manipulation of objects.

Finally, the transition from presential to remote assessment raises several methodological questions. The onset of the pandemic and implementation of restrictions in presential assessment led many researchers to adopt CTA as the default mode of assessment (as the only method that is compatible with safe distancing restrictions), sometimes hastily, and overlooking the issues of adapting presential tests to the online medium. As the context of our research and this paper is in the pediatric domain, it is worth emphasizing the potential issues that hamper the widespread adoption of CTA in pediatric populations. First is the fact that the bulk of CTA is still performed in the context of clinical and non-pediatric populations, so that most published work focuses on adult participants and patients. In this context, there is a risk of overlooking the particularities of CTA in pediatric populations, such as more variable attentional capacity, and higher susceptibility to fatigue and disengagement boredom. Another factor is the fact that CTA is a relatively novel methodology that keeps evolving in synchrony with the development of the underlying technology, so the main themes and issues are not exhaustively mapped yet. Considering these issues, the present paper aims at identifying solutions to the above-mentioned shortcomings in CTA by understanding its evolution and issues in the context of experimental research. A scoping review methodology (Munn et al., 2018) is used to examine the different technical, methodological, and ethical themes/issues in CTA in experimental research in the last two decades, with an emphasis on the typically developing pediatric population, although given the volume of non-pediatric CTA publications, relevant articles in the broader tele-medicine field are also discussed. In addition, the present study aims to map the growth in research, as measured by number of publications across the years by topic, to identify the main issues in pediatric CTA.

## 2 Methods

### 2.1 Classification of articles and inclusion criteria

To address the main themes and issues, articles that explicitly addressed pediatric CTA were chosen and classified as “directly relevant.” Articles were deemed to be directly relevant if they were empirical, review or meta-analysis articles published in peer reviewed journals written in English, using samples of participants aged 18 years old or below and if they addressed remote evaluation of one or more cognitive functions, diagnostic assessment of psychological/psychiatric disorders, performance of children/adolescents in educational settings or evaluation of instruments for pediatric remote cognitive assessment.

In addition, to examine the general evolution and adoption of CTA, articles with a broader scope within the domain of remote cognitive assessment were selected, although these were not necessarily on pediatric populations. These articles were classified “indirectly relevant.” Articles were deemed to be “indirectly relevant” if they did not meet the requirements to be classified as “directly relevant” but were articles on psychological, psychiatric, or neurodevelopmental assessment, both remote and self-administered, self-administered interventions using computerized tools (desktop, mobile or wearable apps) that were or could be adapted for remote use or addressed the assessment of speech, hearing and language disorders with at least a cognitive component (i.e., not exclusively physical such as cleft palate).

For both the “directly” and “indirectly” relevant categories, articles were excluded if they were book chapters, conference papers or commentaries, or other articles not published in peer reviewed journals, in languages other than English, or if they addressed studies on medical, health or educational professionals’ tele-training effectiveness or policies, or surveys on consumer/patient acceptability or views on remote intervention or assessment.

### 2.2 Search strategy

**Figure 1:**
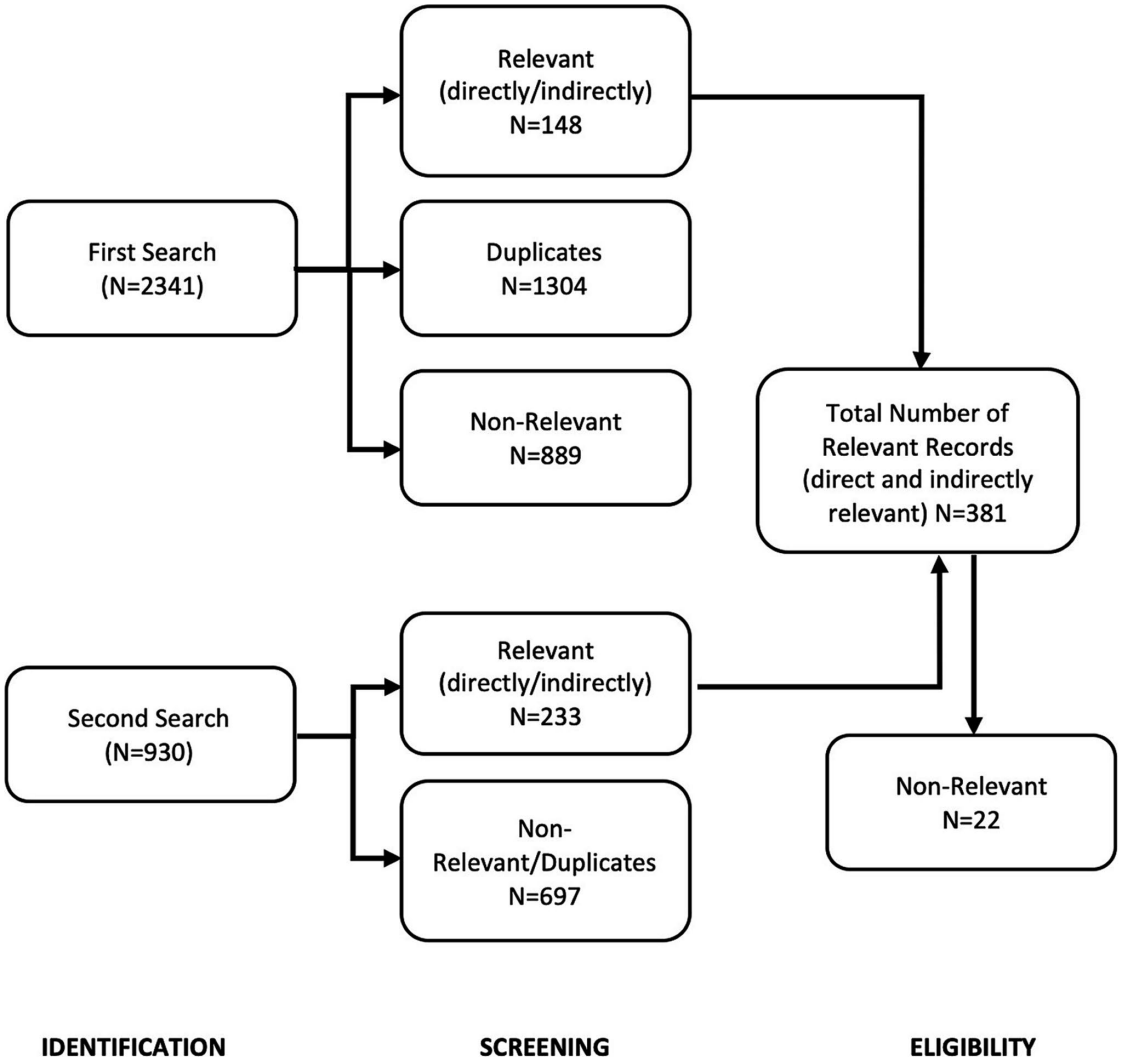
Flowchart representing the number of publications in each step of the PRISMA protocol for studies that included participants aged 18 and below.

Article identification and classification was performed following the PRISMA (Moher et al., 2009) protocol for scoping review processes, through four stages, namely: Identification, Screening, Eligibility and Selection of Included studies (see Figure 1).

### 2.3 Identification and screening

We performed a preliminary initial search to identify the most common text words in the title and abstract of the papers, as well as the index terms. These were identified as:

*“online,” “internet,” “remote,” “assessment,” “test,” “experiment,” “survey,”*

*“cognitive,” “cognition,” “behavior/behaviour,” “behavioral/behavioural,”*

*“therapist,” “experimenter,” “researcher,” “guided” and “assisted” and “interactive.”*

After keywords were identified, The PsycArticles, PsycInfo, PubMed, Scopus and Web of Science databases were queried in August 2021. The search was restricted to articles published between 1/1/2000 and 31/8/2021. The search was conducted with the “title” and “abstract” fields, utilizing search terms composed by the abovementioned keywords joined by OR and AND Boolean operators:

[“online OR internet OR remote”] AND [“assessment OR test OR experiment OR survey”] AND [“cogniti* OR behavio*”] AND [“therapist OR experimenter OR researcher”] AND [“guided OR assisted OR interactive”]. To obtain a more comprehensive overview of the literature, a second search was conducted on 24/3/2022 on Google Scholar, this time using the search term: “*[remote assessment] AND [cognitive abilities] AND [children]*.” The search was restricted to articles published between 1/1/2000 and 24/3/2022.

## 3 Results

### 3.1 Article search results

As mentioned earlier, the PRISMA protocol was used in order to select relevant papers for the review. Here, papers were examined in four stages (Identification, Screening, Eligibility and Selection of Included studies.).

In the identification stage, all papers queried from database searches were included: The first search yielded 2,341 records, and the second yielded 930 records, thus a total of 3,271 articles were identified. In the screening stage, 1,308 duplicate articles were identified and removed (1,304 for the first query and 4 for the second). Articles not in English or not published in peer reviewed journals were removed (889 for the first search and 697 for the second search), thus a total of 381 records remained (148 from the first query and 233 from the second, see Figure 1).

In the eligibility stage, the abstracts of the 381 records were screened independently for relevance by three researchers (N.V.G, P.S.Q.C., and M.L.M.Y), who noted the main theme of the article. Researchers agreed on 359 out of 381 records (94.22% agreement rate). After discussion, 22 records were deemed non-relevant and were discarded. The researchers jointly discussed the relevance of the remaining 359 records, and their classification as directly or indirectly relevant articles with a 100% agreement rate (for lists of all papers that were included in the classification see Supplementary Table 1). Finally in the selection stage, 301 records were classified as indirectly relevant and 58 as directly relevant.

Furthermore, based on the topic of the abstracts of all articles, researchers identified different themes independently. Overlap in themes was very prevalent in articles comparing remote and presential instruments, random trials/pilots/effectiveness studies, and articles on the development of instruments for remote assessment. In the absence of norms for novel remote assessment instruments, studies often described the development of the instrument, together with a comparison (in terms of scores) with its presential equivalent to gauge its effectiveness. Upon comparison and further discussion of the list of themes of each researcher, a consensus was reached for a classification using the themes as listed on Table 1: (1) Articles on pilot studies, effectiveness, and random trials, (2) Articles on validation and comparison of remote assessment tools with their presential equivalents, (3) Articles describing guides, study protocols and the development of instruments, (4) Articles on the technical and usability aspects on remote assessment tools, (5) Articles on tele-educational assessment, (6) Articles on ethics/data management in remote assessment, (7) Reviews and meta-analyses on remote assessment, (8) Articles on self-assessment tools.

**Table 1:** Guidelines for classification of articles in the themes identified in the literature.

*Pilots, Effectiveness or Random trials (N = 84)*	Studies that tested the psychometric properties of an existing online assessment tool, without directly comparing it to its presential equivalent
*Validation and remote/presential comparisons (N = 45)*	Studies that performed direct comparisons between the psychometric properties of tests (or other aspects such as feasibility of application, outreach, etc.), presential and online versions of an existing assessment tool.
*Guides/Protocols/Instruments (N = 57)*	Studies describing the experimental design and development of novel online assessment tools, with or without assessment of their effectiveness.
*Technical/Usability (N = 33)*	Articles that describe the technical aspects in the design and development of an assessment tool (but not very specific aspects such as implementation), as well as usability and engagement issues.
*Tele- education (N = 27)*	Articles that address issues experienced by students in their learning, but not studies on teacher training or teacher/parental attitudes toward tele-assessment.
*Ethics/data management (N = 5)*	Articles that address participants’ data privacy and confidentiality, as well as equity and inclusivity but not legal frameworks.
*Theoretical/Reviews/Meta-analyses (N = 96)*	Articles broad in scope that intend to map the main issues in remote cognitive assessment.
*Self-Assessment/self-help (N = 12)*	Studies that describe digital self-assessment/self-help tools such as online/web/app, surveys, which can be self-administered, not exclusively remotely, which typically are used in conjunction of other psychometric tools (e.g., language background questionnaire) but also as assessment tools for intervention (e.g., logging alcohol use in adolescents).

### 3.2 Identification of themes and mapping the growth of publications by year

**Figure 2:**
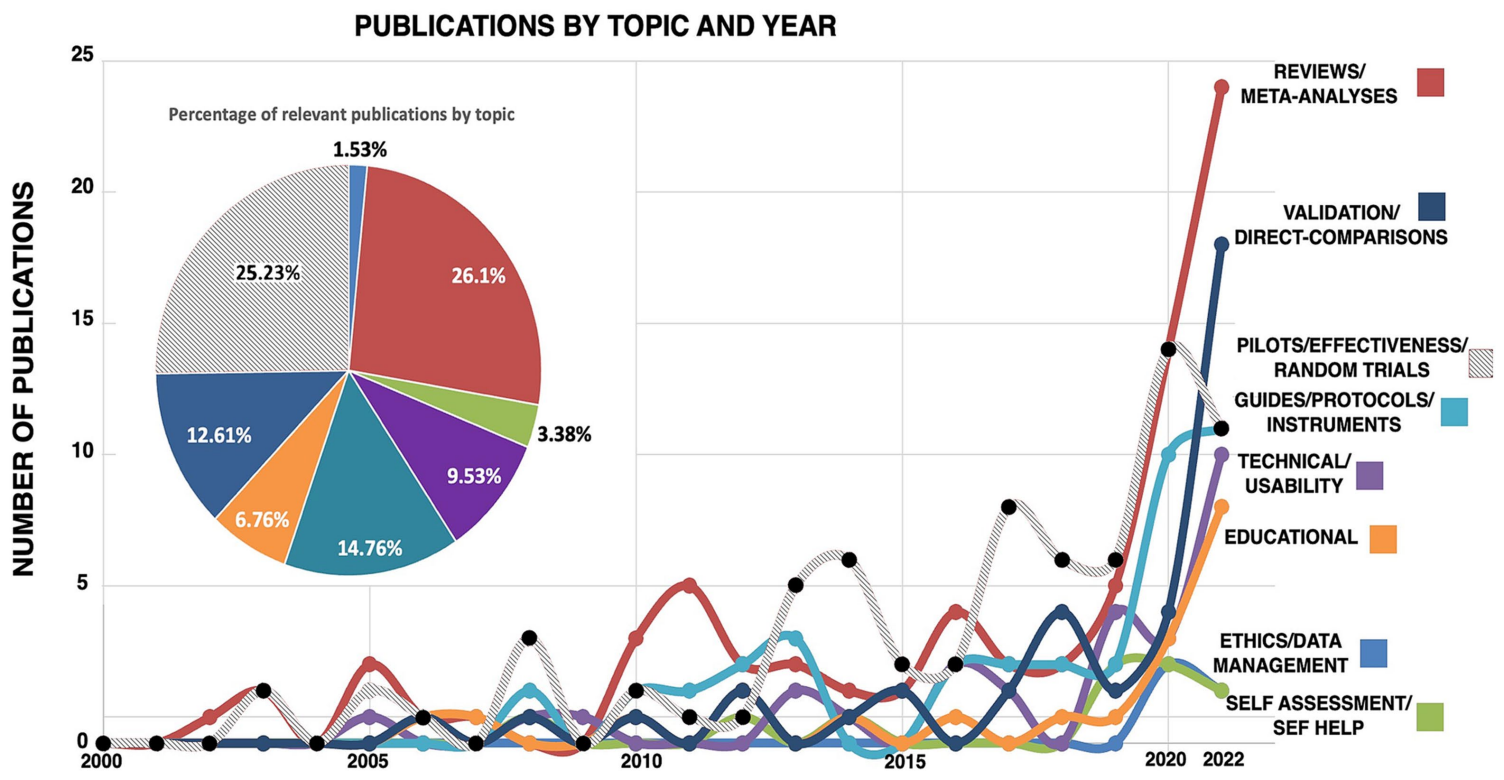
Mapping of publications by year and theme. The top left pie chart indicates the proportion of articles published in each topic between the years 2000 and 2021. The line graph shows the evolution of the number of papers published in each topic from 2000 to 2022.

After classification, the number of publications in each theme was counted, and plotted in Figure 2. The number of publications under each theme was taken as a proxy of the theme’s prevalence for each year between 2000 and 2021 (see Figure 2).

The mapping of the evolution of publications in CTA revealed a steady increase in all themes from 2000 to 2019, and a sharp increase in publications in all themes except for ethics/data management and self-assessment articles after 2019. Between the years 2000 and 2005, most publications were reviews/meta-analyses, and pilot studies/effectiveness/random trials, likely a sign that there was a considerable number of publications already by year 2000, so that conducting meta-analyses and assessing study effectiveness was feasible. It is also possible that the pace at which the internet was developing prior to the dot-com crash in 2000 spurred several meta-analysis papers on the possibilities of tele-medicine. Surprisingly, the number papers of on ethics and data management and self-assessment increased, but at a much lower rate than the other themes around 2018–2019.

### 3.3 Identification of issues in CTA

#### 3.3.1 Technical issues in cognitive tele-assessment

Despite the benefits of CTA, the recent surge in adoption of remote online testing has highlighted its potential shortcomings. One of the most documented issues here concerns technical problems. Whereas technology has matured considerably in the last 20 years, authors report frequent problems due to glitches or lag in the internet connection, software/hardware freezes and crashes (Jaffar and Zehra Ali, 2021), and problems with audio such as echo, low voice, low quality microphone or feedback, or low volume (Jones et al., 2001). More subtle technical problems include inconsistent timing due to the variety in hardware used by participants, thus requiring experimenters to verify whether the participant’s input peripherals conform to the 120 Hz polling rate of the USB standard, whether the participant is using a monitor with a suitable resolution and size for the visual angle that the researchers intended, and so forth (Anwyl-Irvine et al., 2020).

Other technical factors are less tied to the technical capacity of the hardware and more to the variability of the hardware-software combinations that support the online assessment tools. It has been argued that the user experience offered by the online assessment tool emerges from the interaction of the server that hosts the experiment, the speed at which the data is delivered by the internet service provider, the operative system and the browser and code that run on the device. In addition to these combinations, there is a myriad of possible hardware configurations (memory, processing power, whether the participant is using a mobile or desktop device) that can determine the user experience (Anwyl-Irvine et al., 2020).

Additionally, different online formats might involve different technical requirements. For instance, streaming a video that includes important, small resolution details might require high-bandwidth, whereas low-lag might be less important. On the contrary, a video conversation can take place over a lower bandwidth connection but requires very low lag (Rhodes et al., 2020).

Finally, it is apparent that even under optimal software/hardware configurations, online assessment is better suited to certain cognitive domains, such as language tasks, than others such as visuo-motor integration and manual manipulation in intelligence tests (Luxton et al., 2014; Ruffini et al., 2022).

#### 3.3.2 Methodological issues affecting the feasibility of test administration

There is relative consensus that technical problems aside, the most challenging aspect of online cognitive assessment is the fact that experimenters do not have control over the testing environment (Luciana and Nelson, 2002; Garrisi et al., 2020; King et al., 2020). Experimenters might find it difficult to manage children’s behaviors that are not conducive to assessment, such as children’s tendency to fidgeting and using the touchscreen compulsively (Garrisi et al., 2020). Moreover, potential sources of distraction cannot be fully controlled, such as notifications from nearby phones, error notifications from the operative system, or family members interacting in the background (Rhodes et al., 2020; Jaffar and Zehra Ali, 2021). Also out of the experimenter’s control is the possibility that participants might use external aids such as dictionaries, calculators, or search engines (Engelmann et al., 2013; Warneken and Tomasello, 2013).

A special consideration when working with pediatric populations is the fact that children are more variable in terms of attentional capacity, motivation, stamina, and engagement than adults (Hodge et al., 2019; Salinas et al., 2020; Manning et al., 2021; Ruffini et al., 2022), and therefore more susceptible to “Zoom” or virtual fatigue (Bailenson, 2021), and eyestrain over lengthy assessments (Jaffar and Zehra Ali, 2021). These factors not only can reduce assessment performance but can also hamper the possibility of establishing a standardized testing protocol suitable for a larger range of pediatric samples.

A more recent methodological issue in online testing is that the sudden shift to online assessment during the COVID-19 pandemic has pushed experimenters to move their presential testing to the online medium. In many cases, these researchers lack adequate training and familiarity with the procedures of administering online tools, and with rapport-building skills with children (Glueckauf et al., 2018; Farmer et al., 2020; Krach et al., 2020), and this often translates into a less motivating experience for the child, leading to subpar performance.

#### 3.3.3 Equivalence of in-person and online assessment tools

Successful cognitive assessment requires that valid and reliable instruments be used, both in presential and online formats. However, many online tests are “adapted” from tests that were originally intended for presential use (Krach et al., 2020), by altering both the task (procedure, stimuli presentation, adding or removing tasks and so forth), the training of experimenters who will administer the test (Wright, 2020), and the data obtained. In these cases, the validity and reliability of presential tests and their online versions are not directly equivalent (Luxton et al., 2014; Bartram, 2015; Farmer et al., 2020; McGill et al., 2020). In addition, within the online format, validity in one medium (e.g., websites) does not imply validity in another (e.g., mobile/tablet apps) (Bush et al., 2013).

Another factor that requires consideration is whether the test is “unmoderated” (self-administered), or experimenter guided. Unmoderated research has been defined as remote research where participants do not interact with the researchers at all, which is different to approaches that rely on video-conferencing (Rhodes et al., 2020). Tasks that are well suited to unmoderated research are, for instance, those requiring the child to choose pictures corresponding to stories, or tasks that require parent–child interaction are well suited to self-administered testing (Rhodes et al., 2020), but other testing requires behaviors that cannot easily be captured on videoconference, −such as motor, or balance testing (Sheskin and Keil, 2018)- or it requires the experimenter to reengage the children when they lose interest in the task.

#### 3.3.4 Psychometric properties of online assessment tools

Whereas equivalent validity and reliability between online and presential tests are crucial for successful online assessment, most of the information available on the psychometric properties of tele-health-based assessments are based on norms extracted from the equivalent presential procedures (Luxton et al., 2014), usually from clinical studies on adults (Hyler et al., 2005; Brearly et al., 2017; Farmer et al., 2020). In order to compare online and presential tests, most studies use measures of inter-rater reliability, test–retest correlations and effect size (such as Cohen’s *d*) between versions.

The literature describes overall equivalence -but with some discrepancies- between presential tasks and their online equivalents. For instance, Ruffini et al. (2022) found inter-rater agreement and statistical correlations between presential and online scores in 23 studies on clinical and typically developing pediatric participants in the domains of language (*N* = 11 studies), verbal short and long term memory (*N* = 5), intelligence (*N* = 5), academic abilities (*N* = 4), neuropsychological functions (*N* = 6), communication and social interaction abilities (*N* = 1) and quality of life, psychiatric symptoms and social and occupational functioning (*N* = 1).

Similarly, test equivalence as indexed by low inter-rater variability has been reported in tests of IQ, language, and academic in children with cochlear implants (Stain et al., 2011), executive functions, verbal abilities, working memory, motor-free processing speed and visuo-motor integration (Harder et al., 2020), verbal fluency (Ragbeer et al., 2016), and speech and language tasks, such as judgment of speech sounds, detection of plural forms and phonetic discrimination in similarly sounding words (Taylor et al., 2014). It is worth noting that there are discrepancies in the literature: for instance, some studies report agreement in online and presential tests of speech articulation (Luciana and Nelson, 2002) while others do not (Taylor et al., 2014). Likewise, some authors report agreement in working and long memory tests (Luciana and Nelson, 2002; Harder et al., 2020) while others report no agreement in phonological memory tests (Werfel et al., 2021), and no agreement in the digit span backwards working memory test (Stain et al., 2011).

In addition to test–retest scores and inter-rater reliability, several studies have used effect size (ES) to assess study equivalence. An early meta-analysis of 14 studies comparing in-person psychiatry and tele-psychiatry (Hyler et al., 2005) found no differences in effect size between the two modalities. In that study, effect sizes were calculated for studies that provided measures that could be converted into Pearson’s *r*, such as χ^2^, cross-tabulations that could be converted to χ^2^, *z-*scores, mean and SD (which were transformed to Cohen’s d, and subsequently converted to *r*), and P and n (converted first to t-scores, then to *r*). Studies using inter-rater reliability, kappa coefficients or Interclass Correlation Coefficients (ICC) which could not be converted into ES measures were analyzed separately (Hyler et al., 2005). Turning to assessment in non-clinical settings, such as in the educational context, negligible differences in effect sizes between online and presential versions have been reported for the Woodcock Johnson test of academic achievement (Wright, 2018), and the WISC-V intelligence test (Wright, 2020). To assess equivalence of the WISC-V test Wright (2020) used a case–control matched design where the two participant groups were matched on age and gender and each group only received the test in one format. Equivalence was assessed using Cohen’s *d* and a two one-sided *t*-test (TOST), which is a more conservative *t*-test analysis based on the smallest effect size of interest (SESOI) rather than confidence interval bounds (Schuirmann, 1987; Seaman and Serlin, 1998; Lakens, 2017). However, it has been argued that this design informs on the equivalence between mean scores of both (online and presenting) groups, but that a within-group design is required to examine whether an individual would obtain the same score in online and presential formats (Farmer et al., 2020). The TOST has also been used to assess the online/presential equivalence of the picture rotation test for spatial ability (Quaiser-Pohl, 2003), where the difference in *z*-scores between presential and online versions was less than 1 SD from 0 (Bambha and Casasola, 2021). In the same study, authors calculated the internal validity (Cronbach’s alpha) of online and presential versions of the task and found no differences in validity, but presence of more extreme residuals in the online version suggested that children became distracted, fatigued, or less engaged in the online version of the task, and thus performed worse than it was expected for their age group. Besides the TOST, the Mann–Whitney U test (the nonparametric equivalent of the *t*-test) has been used to assess equivalence between online and presential versions of tests. For instance, in a study on the effectiveness of online and presential versions of tele-neuropsychological intervention in pediatric demyelinating disorder patients, the Mann–Whitney *U* test showed no differences in effectivity between online and presential tests for language, memory, visual perception, visuomotor integration and math fluency (Harder et al., 2020). Lastly, it has been noted that there is a general lack of guidelines for cognitive assessment of children in terms of parameters of assessment, feasibility of cognitive functions that can be assessed, personnel requirements and so forth (Luciana and Nelson, 2002; Edwards et al., 2012), although the current article mapping shows that this situation is changing, with a steep rise in the publication of guidelines after 2020 (see Figure 2).

#### 3.3.5 Ethical issues in cognitive tele-assessment

Online cognitive assessment poses ethical issues that have been previously described in detail in the tele-medicine literature. For instance, the APA (American Psychological Association) emphasizes that tele-health practices should adhere to the same ethical standards as standard, in-person psychology. By extension, online assessment should maintain the same ethical standards as presential assessment. However, the format of tele-assessment can make several procedures more complex. For instance, obtaining informed consent might be less straightforward than in presential settings, so this process needs to be especially thorough and transparent, where experimenters are in contact with participants in order to clarify any issues that might arise, and take additional steps to ensure validity and reliability of the instruments (such as carrying out scientific validation studies prior to using novel instruments), and ensure that these are adequate for the population being assessed (American Educational Research Association et al., 2014; American Psychological Association, 2020), as well as to be explicit about their limitations and what measures are in place to protect the patient or participant’s privacy and electronic data (Luxton et al., 2014).

In addition, there is general consensus that the shift to digital transmission of participant data in online assessment has increased the potential for infringement of data privacy and confidentiality (Hyler and Gangure, 2004; Clark et al., 2010) and in this context, experimenters have the duty to safeguard patients or participants’ rights to privacy, security and confidentiality. Hyler and Gangure (2004) describe Privacy as “*an individual’s claim to control the use and disclosure of personal information*,” Security as “*the safeguards in an information system that protect it and its contents against unauthorized disclosure, and limit access to authorized users in accordance with established policy*,” and Confidentiality as “*the status accorded to information that indicates it is sensitive for certain reasons and must therefore be protected and access to it controlled*” (Hyler and Gangure, 2004, p. 274).

Besides data privacy and handling, another ethical issue in CTA is equity and accessibility. Whereas it is widely acknowledged that one of the main benefits of online assessment is that it can lower the barriers of access (for instance, for participants in remote rural communities, or patients with rare disorders who cannot attend in-person assessment), remote assessment requires that participants have access to suitable technology, and disparities in access can exacerbate issues of inequity (American Psychological Association, 2020). For instance, access to suitable devices, high speed internet might be unavailable to participants living in remote sites, even in developed countries (Houston et al., 2012), suitable household environment for assessment might be restricted to participants with a higher socio-economic background and there are still disparities in network infrastructure between developed and developing countries (Jaffar and Zehra Ali, 2021).

## 4 Discussion

As mentioned at the outset, the main factor differentiating remote from presential cognitive assessment is the use of online tools instead of an experimenter who is physically present in order to deliver the assessment instrument. Remote assessment is associated with logistic benefits such as reduced time and travel costs for both participants and experimenters, access to remote, demographically diverse and larger samples, or the possibility of observing participants in their home (e.g., in studies that examine parent–child relations) (Rhodes et al., 2020). In addition, remote online assessment has been shown to facilitate the scheduling of repeated sessions, to facilitate multi-site collaborations (Ebersole et al., 2016), and to be more conducive to longitudinal research, by enabling following-up on participants who have moved home or school (Hodge et al., 2019). Online assessment also facilitates the standardization of protocols for study replicability (Pashler and Wagenmakers, 2012; Open Science Collaboration, 2015; Scott and Schulz, 2017). Despite these advantages, CTA in children is susceptible to technical issues (hardware and software related), issues pertaining to pediatric populations (reduced attentional stamina, tendency to distraction and fidgeting), methodological issues (relating the online adaptation and administration of instruments traditionally used in face-to-face settings) and ethical issues (privacy, confidentiality and data protection and handling). In the following sections, we discuss and propose solutions to the issues identified in the literature search.

### 4.1 Technical issues in cognitive tele-assessment

Many of the shortcomings in CTA concern technical issues, and as such, a number of strategies can be adopted to address these. Besides problems that are solved as new technologies emerge or improve (e.g., improvement in latency and resolution in videoconferencing systems), inconsistencies due to hardware/software combinations can be mitigated by limiting the choice of software platforms that participants can use to access experimental materials. Experimenters might, for instance, require participants to use only a mouse as input method on laptop or desktop computers (no touchscreens or touchpads), in order to eliminate potential discrepancies in the scores of participants using different types of input devices. In addition, technical checks can be scheduled to ensure that participants’ internet speed connection and audio volume levels are adequate.

As a fallback solution in case of serious technical problems, presential testing sessions can be scheduled, where assessment materials are identical to those used online, for instance, participants might access online materials through a computer provided by the experimenter.

#### 4.1.1 Methodological issues affecting the feasibility of test administration

Online testing is characterized by the fact that the experimenter has little -if any- control over the testing environment, so testing sessions are especially susceptible to potential distractions or other elements outside of the experimenter’s control. One way of countering these factors is for the experimenters to request that parents/caregivers help with managing the child’s behavior. Whereas parental assistance is beneficial, instructing the parents increases the additional workload of the experimenter, and has the potential of lengthening the testing session and introducing an “observer effect” where parents might influence their children’s responses just by being present in the same room or might even actively try to help their children (Engelmann et al., 2013). Mitigating these issues might require dedicating additional time for experimental set-up, explaining instructions, and training the parent/caregiver to help in controlling the child’s behavior and motivation.

Children have typically lower attentional capacity and stamina compared to adults, and the nature of cognitive tests, which are typically boring, tiring and repetitive for children (Lumsden et al., 2016) can result in the child disengaging from the task and negatively impacting the quality of the data collected.

An emphasis in rapport building with the child, as well as making the tasks more engaging can help minimize these issues. For instance, experimenters can turn to present online assessment tools as multi-sensory, interactive media (providing video, sound, feedback) to increase engagement and participation (Luciana and Nelson, 2002), or to implement more elaborate game-like features such as storytelling or competitive elements (points systems, leader and score boards) while providing direct feedback to increase motivation and to reduce participant dropout over prolonged or multiple testing sessions (Lumsden et al., 2016). However, it has been noted that introducing gamified elements to online cognitive assessment while maintaining scientific validity is a challenging process, and that some game mechanics can have a detrimental effect on performance. For instance, online games that are well structured, with clearly defined goals and that provide direct feedback throughout the task can artificially enhance performance in children with ADHD, thus which would otherwise not occur in presential settings or with formal tests (Lumsden et al., 2016).

#### 4.1.2 Equivalence of in-person and online assessment tools

As discussed previously, adapting presential versions of tests for their use online does not guarantee that reliability and validity will be equivalent between modalities, so it is important to use tests that are well documented in terms of reliability and validity (American Educational Research Association et al., 2014; Bartram, 2015). However, when online equivalents of well-documented tests are not available, experimenters are advised to adapt existing test taking into consideration whether the experimental format is suitable for the online medium. In particular, the presence of a proctor (e.g., when tests are guided by an experimenter rather than self-administered) (Wright, 2018) is essential where assessment is conducted through videoconference (Brearly et al., 2017), but can also be beneficial in the case of self-administered tests, web screeners of cognitive assessment in ASD, ADHD and emotional dysregulation in preschoolers (Baker et al., 2020), and cognitive testing for psychological assessment and psychotherapy (Heesacker et al., 2020).

### 4.2 Psychometric properties of online assessment tools

Successful CTA relies on instruments with adequate reliability and validity. However, during the sudden push for CTA adoption during the pandemic, many tests were adapted for use online, based on validity and reliability norms of their presential equivalents, and without clear motivation on what measures should be used to establish equivalence between presential and online test versions or what cognitive functions can be assessed and how to train experimenters administering the tasks. A better option in these situations is that experimenters attempt to ensure adequate equivalence between presential and online tests, by using both standardized effect size measurements (to allow for comparison across different studies), as well as a number of correlations between online and presential test versions, such as test–retest scores, internal validity scores (Cronbach’s alpha), or parametric (e.g., TOST) and non-parametric (e.g., Mann–Whitney U) means comparisons. In addition, ensuring presential/online equivalence in pediatric populations requires that the nature of the test be adapted to the online format (e.g., avoiding tests that involve manual manipulation or tasks that children might find tiring in the online medium).

### 4.3 Ethical issues in cognitive tele-assessment

Whereas CTA should strive to protect participants’ rights to privacy, security and confidentially in the same way as presential assessment, data handling issues might arise due to the fact that personal information is transmitted through the internet. Again, there is still a lack of consensus in procedures for data handling during CTA, and regulations vary greatly depending on the geographical location of the assessment. Regardless of modality (presential or online), our current projects are subject to the ethical standards of Nanyang Technological University’s Institutional Review Board. However, in projects involving remote assessment, data management is governed by Nanyang Technological University’s research data policy,^1^ as well as the Singapore Personal Data Protection act,^2^ which largely (a) recognize the right of individuals to protect their personal data and the need of organizations to collect, use or disclose personal data for purposes that a reasonable person would consider appropriate in the circumstances, and (b) permits the researcher to retain a copy of the data generated during research in equipment where the university has full access and control for 10 years after publication or completion of the project, and mandates that data be protected from loss, theft, damage and unauthorized access. Moreover, data collection in our research is supported by the Gorilla.ac web hosting and email sending services, with web hosting relying on Microsoft Azure servers located in Ireland, EU^3^ and file storage relying on Dropbox,^4^ which follow the pertinent EU and US regulations. Specifically, Gorilla.sc specify that any data sent from and to Gorilla is encrypted with the Transport Layer Security Protocol (TLS/SSL) and is GDPR compliant, no IP addresses are collected, and the researcher has ownership and full control of the data collected. Data collected is anonymous and identifiable only by private ID numbers.^5^,^6^

Besides data privacy and confidentiality issues, another aspect of CTA that requires examination is the ethical principles of inclusiveness, justice and fairness, which highlight the right of participants in getting recognition and benefits from the research (American Psychological Association, 2020). In particular, as participation in CTA-based research requires access to suitable equipment, ensuring equal chance for recruitment is paramount in order to avoid excluding participants who do not have access to suitable equipment, and to avoid introducing a selection bias in the study.

As mentioned earlier, one possible solution researchers can adopt in order to include participant who might otherwise be unable to join the experiment is to host presential sessions and provide suitable equipment to access the online materials. At an institutional level, the Singapore government started supplying secondary schools with personal learning devices in 2020, to provide all students in Singapore with a school-issued laptop or a tablet by 2024^7^ that can be used for home-based learning.

## 5 Conclusion and future directions

The body of literature examined in the present review suggests several trends in the field in the coming years. First, it is likely that refinements in the technology that supports CTA will be accompanied with reduced costs and reduced potential for technical problems. These factors in turn might enable access to larger and more diverse samples, increase in the number of longitudinal studies and a shift toward the development of future assessment instruments that use the remote modality by default, and that equivalence between remote and online versions is assessed more rigorously than currently (e.g., by piloting both versions, not just referring to equivalence scores). Developing new instruments will also require developing protocols for their administration. There might also be an increase in research toward implementing more engaging paradigms for online tests, with instruments for child assessment adopting gamified approaches, and also new paradigms, such as virtual reality. In contrast to these developments, three factors are likely to prevent change in the field. First is the limited nature of children’s attentional abilities, second the inability of the researcher to control the testing environment and third the process of informing and obtaining informed ethical consent from the caretakers (Luciana and Nelson, 2002; Garrisi et al., 2020; King et al., 2020). In order to mitigate these factors, based on our experience, steps to ensure participant compliance can be adopted: First, on the technical side, careful scrutiny of the costs and benefits of the different available platforms and devices that can be used to access the test materials, as well as a scheduling of a technical test session. Second, is the training of experimenters in online test administration, as well as solving technical issues during set-up and testing, and in rapport building with children and parents. Based on empirical observations in the researchers’ current projects, data quality and participant engagement are typically higher when parents show an earnest interest in the project (e.g., by asking questions, being proactive and discussing with the experimenters any potential issues with the child or the testing environment within the household). Third, testing sessions can benefit from parental assistance, after providing the parent with clear instructions on their role, how to manage technical issues and, if required, how to explain the task instructions to their children in terms that they understand. A fourth recommendation is that cognitive tasks be presented in a gamified format, preferably as subtasks embedded into a narrative with a larger, main goal. With this format, even very different tasks seem related rather than disjoined, and completion of one task motivates the participant and brings them closer to accomplishing the main goal.

Finally, researchers are advised to implement careful data management practices. As cybersecurity threats increase with the spread of adoption of online assessment technologies, a more robust approach to data handling will be required, for instance, by using encrypted connections and data storage and two-factor authentication process for access to the test materials. As such, it is likely that there will be a large increase in the volume of publications on ethics and data management policies for CTA in the coming years.

## Data availability statement

The original contributions presented in the study are included in the article/Supplementary material, further inquiries can be directed to the corresponding author.

## Author contributions

NV-G: Conceptualization, Methodology, Project administration, Supervision, Visualization, Data curation, Investigation, Writing – original draft. PC: Data curation, Investigation, Methodology, Writing – original draft. MY: Data curation, Investigation, Methodology, Formal analysis, Writing – original draft. C-YW: Conceptualization, Methodology, Project administration, Writing – review & editing. SC: Conceptualization, Funding acquisition, Methodology, Project administration, Supervision, Visualization, Writing – original draft, Writing – review & editing.

## Footnotes

^1^https://www.ntu.edu.sg/research/ntu-research-data-policy

^2^https://sso.agc.gov.sg/Act/PDPA2012

^3^https://azure.microsoft.com

^4^www.dropbox.com

^5^https://support.gorilla.sc/support/due-diligence#internalreviewboardsethics

^6^https://app.gorilla.sc/privacy

^7^https://www.moe.gov.sg/microsites/cos2020/refreshing-our-curriculum/strengthen-digital-literacy.html
