# Supplementary material for: Cognitive pediatric tele-assessment: a scoping review
Source: Front Psychol. 2023 Dec 15;14:1288021. doi: 10.3389/fpsyg.2023.1288021 (PMC10754967; doi:10.3389/fpsyg.2023.1288021)
Supplement: Supplementary file 1 [file Table_1.docx]

Supplementary Material

Cognitive Pediatric Tele assessment: A Scoping Review.

Nestor Vinas-Guasch^15^, Phoebe Chia Si Qi^1^, Michelle Yap Li-Mei^2^, Chiao-Yi Wu^23^, and S.H. Annabel Chen^1234^

**** Correspondence:** Annabel S.H. Chen. annabelchen@ntu.edu.sg

## Supplementary Table 1: List of publications used in the "relevant" group.

|  | |  |  | | ***Articles on Pilot Studies, Effectiveness or Random trials*** | | |  | |  |
| --- | --- | --- | --- | --- | --- | --- | --- | --- | --- | --- |
| 1 | |  |  | | Outcomes of an integrated telehealth network demonstration project | | | S. Dimmick et al. | | 2003 |
| 2 | |  |  | | Evaluating the efficacy of tele-cognitive rehabilitation for functional performance in three case studies | | | S. F. Tam et al. | | 2003 |
| 3 | |  |  | | A pilot study on the effectiveness of tele-analogy-based problem-solving training for people with brain injuries | | | W. Soong et al. | | 2005 |
| 4 | |  |  | | Putting the Pieces Together: Preliminary Efficacy of a Web-Based Family Intervention for Children with Traumatic Brain Injury | | | S. L. Wade et al. | | 2005 |
| 5 | |  |  | | Efficacy of internet therapy for panic disorder | | | B. Klein et al. | | 2006 |
| 6 | |  |  | | The climate sadness program of internet-based treatment for depression: A pilot study | | | S. Perini et al. | | 2008 |
| 7 | |  |  | | Predicting successful treatment outcome of web-based self-help for problem drinkers: Secondary analysis from a randomized controlled trial | | | H. Riper et al. | | 2008 |
| 8 | |  |  | | Web-based self-help for problem drinkers: A pragmatic randomized trial | | | H. Riper et al. | | 2008 |
| 9 | |  |  | | The climate panic program of internet-based treatment for panic disorder: A pilot study | | | E. Wims et al. | | 2008 |
| 10 | |  |  | | Motivating the unmotivated for health behavior change: a randomized trial of cessation induction for smokers | | | M. J. Carpenter et al. | | 2010 |
| 11 | |  |  | | A therapist-assisted cognitive behavior therapy Internet intervention for posttraumatic stress disorder: Pre-, post- and 3-month follow-up results from an open trial | | | B. Klein et al. | | 2010 |
| 12 | |  |  | | Internet-Based Treatment of Depression: A Randomized Controlled Trial Comparing Guided with Unguided Self-Help | | | T. Berger et al. | | 2011 |
| 13 | |  |  | | Validity of conducting clinical dysphagia assessments for patients with normal to mild cognitive impairment via telerehabilitation | | | E. Ward et al. | | 2012 |
| 14 | |  |  | | Predictors and moderators of internet- and group-based cognitive behaviour therapy for panic disorder | | | S. E. Alaoui et al. | | 2013 |
| 15 | |  |  | | Effectiveness of guided Internet-based cognitive behavior therapy in regular clinical settings | | | G. Andersson and E. Hedman | | 2013 |
| 16 | |  |  | | Internet-based behavioral activation and acceptance-based treatment for depression: A randomized controlled trial | | | P. Carlbring et al. | | 2013 |
| 17 | |  |  | | Computerized assessment of cognitive late effects among adolescent brain tumor survivors | | | H. Conklin et al. | | 2013 |
| 18 | |  |  | | The Clinical Effectiveness of Web-Based Cognitive Behavioral Therapy With Face-to-Face Therapist Support for Depressed Primary Care Patients: Randomized Controlled Trial | | | R. S. Hoifodt et al. | | 2013 |
| 19 | |  |  | | Internet cognitive behavioural therapy for mixed anxiety and depression: A randomized controlled trial and evidence of effectiveness in primary care | | | J. M. Newby et al. | | 2013 |
| 20 | |  |  | | Internet and patient empowerment in individuals with symptoms of an eating disorder: A cross-sectional investigation of a pro-recovery focused e-community | | | J. J. Aardoom et al. | | 2014 |
| 21 | |  |  | | Guided internet-delivered cognitive behavior therapy for post-traumatic stress disorder: A randomized controlled trial | | | D. Ivarsson et al. | | 2014 |
| 22 | |  |  | | Integrative testimonial therapy: an Internet-based, therapist-assisted therapy for German elderly survivors of the World War II with posttraumatic stress symptoms | | | C. Knaevelsrud et al. | | 2014 |
| 23 | |  |  | | Internet-Delivered Cognitive Behavior Therapy for Adolescents with Obsessive-Compulsive Disorder: An Open Trial | | | F. Lenhard et al. | | 2014 |
| 24 | |  |  | | A randomized controlled trial of an internet-based therapist-assisted indicated preventive intervention for prolonged grief disorder | | | B. T. Litz et al. | | 2014 |
| 25 | |  |  | | Delivering group treatment via videoconference to individuals with traumatic brain injury: a feasibility study | | | T. Tsaousides et al. | | 2014 |
| 26 | |  |  | | International telemedicine consultations for neurodevelopmental disabilities | | | P. L. Pearl et al. | | 2014 |
| 27 | |  |  | | Feasibility study of the BrightBrainer™ integrative cognitive rehabilitation system for elderly with dementia | | | G. Burdea et al. | | 2015 |
| 28 | |  |  | | Efficacy of Cognitive Behavioral Therapy for Insomnia in Adolescents: A Randomized Controlled Trial with Internet Therapy, Group Therapy and A Waiting List Condition | | | E. J. de Bruin et al. | | 2015 |
| 29 | |  |  | | Internet-based cognitive—behavior therapy for procrastination: A randomized controlled trial | | | A. Rozental et al. | | 2015 |
| 30 | |  |  | | Predicting outcome in internet-based cognitive behaviour therapy for major depression: A large cohort study of adult patients in routine psychiatric care | | | S. E. Alaoui et al. | | 2016 |
| 31 | |  |  | | Internet-delivered acceptance-based behaviour therapy for generalized anxiety disorder: A randomized controlled trial | | | M. Dahlin et al. | | 2016 |
| 32 | |  |  | | Internet-delivered cognitive therapy for PTSD: a development pilot series | | | J. Wild et al. | | 2016 |
| 33 | |  |  | | Effectiveness of a Web-Based Intervention in Reducing Depression and Sickness Absence: Randomized Controlled Trial | | | T. Beiwinkel et al. | | 2017 |
| 34 | |  |  | | Twelve-month follow-up of a randomized controlled trial of internet-based guided self-help for parents of children on cancer treatment | | | M. Cernvall et al. | | 2017 |
| 35 | |  |  | | Internet-based cognitive-behavioural writing therapy for reducing post-traumatic stress after intensive care for sepsis in patients and their spouses (REPAIR): study protocol for a randomised-controlled trial | | | R. Gawlytta et al. | | 2017 |
| 36 | |  |  | | From the experience of interactivity and entertainment to lower intention to smoke: A randomized controlled trial and path analysis of a web-based smoking prevention program for adolescents | | | G. E. Khalil et al. | | 2017 |
| 37 | |  |  | | Therapist-guided internet-delivered cognitive-behavioural therapy supplemented with group exposure sessions for adolescents with social anxiety disorder: a feasibility trial | | | M. Nordh et al. | | 2017 |
| 38 | |  |  | | Effectiveness and cost-effectiveness of a guided Internet- and mobile-based intervention for the indicated prevention of major depression in patients with chronic back pain—study protocol of the PROD-BP multicenter pragmatic RCT | | | L. Sander et al. | | 2017 |
| 39 | |  |  | | Efficacy of Seren@ctif, a Computer-Based Stress Management Program for Patients With Adjustment Disorder With Anxiety: Protocol for a Controlled Trial | | | D. Servant et al. | | 2017 |
| 40 | |  |  | | Investigating the accuracy of a novel telehealth diagnostic approach for autism spectrum disorder | | | C. Smith et al. | | 2017 |
| 41 | |  |  | | Extending research on Emotion Regulation Individual Therapy for Adolescents (ERITA) with nonsuicidal self-injury disorder: open pilot trial and mediation analysis of a novel online version | | | J. Bjureberg et al. | | 2018 |
| 42 | |  |  | | Implementation of internet-delivered CBT for children with anxiety disorders in a rural area: A feasibility trial | | | M. Jolstedt et al. | | 2018 |
| 43 | |  |  | | Early identification of ASD through telemedicine: Potential value for underserved populations | | | A. Juárez et al. | | 2018 |
| 44 | |  |  | | Estimating mini mental state examination scores using game-specific performance values: A preliminary study | | | H. Jung et al. | | 2018 |
| 45 | |  |  | | Therapist-Assisted Internet-Based Cognitive Behavioral Therapy Versus Progressive Relaxation in Obsessive-Compulsive Disorder: Randomized Controlled Trial | | | M. Kyrios et al. | | 2018 |
| 46 | |  |  | | Pilot trial of a tele-rehab intervention to improve outcomes after stroke in Ghana: A feasibility and user satisfaction study | | | F. S. Sarfo et al. | | 2018 |
| 47 | |  |  | | Remote detection and Classification of human stress using a depth sensing technique | | | Y. Shan et al. | | 2018 |
| 48 | |  |  | | Ontario health technology assessment series: Internet-delivered cognitive behavioural therapy for major depression and anxiety disorders: A health technology assessment | | | | | 2019 |
| 49 | |  |  | | Reducing Stress and Preventing Depression (RESPOND): Randomized controlled trial of web-based rumination-focused cognitive behavioral therapy for high-ruminating university students | | | L. Cook et al. | | 2019 |
| 50 | |  |  | | Therapist-assisted online treatment for child conduct problems in rural and urban families: Two randomized controlled trials | | | M. R. Dadds et al. | | 2019 |
| 51 | |  |  | | Guided internet-based cognitive behavioral therapy for adolescent anxiety: Predictors of treatment response | | | S. Stjerneklar et al. | | 2019 |
| 52 | |  |  | | Internet-Based Cognitive-Behavioural Intervention for Women with Premenstrual Dysphoric Disorder: A Randomized Controlled Trial | | | C. Weise et al. | | 2019 |
| 53 | |  |  | | Internet-based cognitive behavioral therapy in stepped care for chronic fatigue syndrome: Randomized noninferiority trial | | | M. Worm-Smeitink et al. | | 2019 |
| 54 | |  |  | | Implementation of internet-delivered cognitive behaviour therapy for pediatric obsessive-compulsive disorder: Lessons from clinics in Sweden, United Kingdom and Australia | | | K. Aspvall et al. | | 2020 |
| 55 | |  |  | | A Web-Delivered Acceptance and Commitment Therapy Intervention With Email Reminders to Enhance Subjective Well-Being and Encourage Engagement With Lifestyle Behavior Change in Health Care Staff: Randomized Cluster Feasibility Stud | | | M. Brown et al. | | 2020 |
| 56 | |  |  | | Therapist-Supported Online Interventions for Children and Young People With Tic Disorders: Lessons Learned From a Randomized Controlled Trial and Considerations for Future Practice | | | L. R. Chamberlain et al. | | 2020 |
| 57 | |  |  | | A randomised controlled trial of therapist-assisted online psychological therapies for posttraumatic stress disorder (STOP-PTSD): Trial protocol | | | A. Ehlers et al. | | 2020 |
| 58 | |  |  | | Predicting treatment failure in regular care internet-delivered cognitive behavior therapy for depression and anxiety using only weekly symptom measures | | | E. Forsell et al. | | 2020 |
| 59 | |  |  | | Feasibility of telehealth-delivered home safety evaluations for caregivers of clients with dementia | | | M. Gately et al. | | 2020 |
| 60 | |  |  | | Deep brain stimulation telemedicine programming during the COVID-19 pandemic: treatment of patients with psychiatric disorders | | | Z. Lin et al. | | 2020 |
| 61 | |  |  | | Evaluation of an internet-based intervention for service members of the German armed forces with deployment-related posttraumatic stress symptoms | | | H. Niemeyer et al. | | 2020 |
| 62 | |  |  | | Internet-delivered mindfulness-based cognitive therapy for anxiety and depression in cancer survivors: A randomized controlled trial | | | E. R. Nissen et al. | | 2020 |
| 63 | |  |  | | High- versus low-intensity internet interventions for alcohol use disorders: results of a three-armed randomized controlled superiority trial | | | C. Sundström et al. | | 2020 |
| 64 | |  |  | | Effects of digital cognitive behavioural therapy for insomnia on insomnia severity: a large-scale randomised controlled trial | | | O. Vedaa et al. | | 2020 |
| 65 | |  |  | | A Survivor's Journey: Preliminary efficacy of an online problem-solving therapy for survivors of pediatric brain tumor | | | S. L. Wade et al. | | 2020 |
| 66 | |  |  | | Feasibility of a brief online psychoeducational intervention for women with sexual interest/arousal disorder | | | N. Zippan et al. | | 2020 |
| 67 | |  |  | | Feasibility and acceptability of Lee Silverman Voice Treatment in progressive ataxias | | | A. Lowit et al. | | 2020 |
| 68 | |  |  | | Blended Care-Cognitive Behavioral Therapy for Depression and Anxiety in Real-World Settings: Pragmatic Retrospective Study | | | A. Lungu et al. | | 2020 |
| 69 | |  |  | | Exploring the feasibility of collecting multimodal multiperson assessment data via distance in families affected by fragile X syndrome | | | L. Bullard et al. | | 2021 |
| 70 | |  |  | | Efficacy of therapist-delivered transdiagnostic CBT for patients with persistent physical symptoms in secondary care: A randomised controlled trial | | | T. Chalder et al. | | 2021 |
| 71 | |  |  | | An efficacy trial of therapist-assisted internet-delivered cognitive-behaviour therapy for older adults with generalized anxiety | | | S. L. Jones | | 2021 |
| 72 | |  |  | | Remote Assessment of Depression Using Digital Biomarkers From Cognitive Tasks | | | R. Mandryk et al. | | 2021 |
| 73 | |  |  | | Internet-delivered Mindfulness-Based Cognitive Therapy for anxiety and depression in cancer survivors: Predictors of treatment response | | | E. R. Nissen et al. | | 2021 |
| 74 | |  |  | | Disturbed sleep as a clinical marker of wish to die: a smartphone monitoring study over three months of observation | | | A. Porras-Segovia et al. | | 2021 |
| 75 | |  |  | | A guided Internet-delivered intervention for adjustment disorders: A randomized controlled trial | | | I. Rachyla et al. | | 2021 |
| 76 | |  |  | | Internet-delivered cognitive processing therapy for individuals with a history of bullying victimization: A randomized controlled trial | | | A. S. Thorisdottir and G. Asmundson | | 2021 |
| 77 | |  |  | | Remote Assessment of Cognition in Kids and Adolescents with Daytime Sleepiness: A pilot study of feasibility and reliability | | | J. Worhach et al. | | 2021 |
| 78 | |  |  | | Pilot study to assess the feasibility of a mobile unit for remote cognitive screening of isolated elderly in rural areas | | | R. Zeghari et al. | | 2021 |
| 79 | |  |  | | mPulse Mobile Sensing Model for Passive Detection of Impulsive Behavior: Exploratory Prediction Study | | | H. Wen et al. | | 2021 |
| 80 | |  |  | | Neurobehavioral dimensions of Prader Willi Syndrome: Relationships between sleep disturbance and psychotic experiences | | | Z. Zhang et al. | | 2021 |
| 81 | |  |  | | Strategies, Recommendations, and Validation of Remote Executive Function Tasks for use with Young Children | | | S. Ahmed et al. | | 2022 |
| 82 | |  |  | | Temporal Speech Parameters Indicate Early Cognitive Decline in Elderly Patients With Type 2 Diabetes Mellitus | | | N. Imre et al. | | 2022 |
| 83 | |  |  | | UCancellation: A new mobile measure of selective attention and concentration | | | A. Pahor et al. | | 2022 |
| 84 | |  |  | | A randomised controlled trial investigating the clinical and cost-effectiveness of Alpha-Stim AID cranial electrotherapy stimulation (CES) in patients seeking … | | | S. Patel et al. | | 2022 |
|  | ***Validation and remote/presential comparisons*** | | | | | | | | | |
|  | 1 | | |  | | A randomized clinical trial study on the effectiveness of a tele-analogy-based problem-solving programme for people with acquired brain injury (ABI) | | | D. W. Man et al. | 2006 |
|  | 2 | | |  | | Predicting treatment outcome in internet versus face to face treatment of panic disorder | | | G. Andersson et al. | 2008 |
|  | 3 | | |  | | Internet administered guided self-help versus individualized e-mail therapy: A randomized trial of two versions of CBT for major depression | | | K. Vernmark et al. | 2010 |
|  | 4 | | |  | | In-person versus telehealth assessment of discourse ability in adults with traumatic brain injury | | | L. Turkstra et al. | 2012 |
|  | 5 | | |  | | Validity of conducting clinical dysphagia assessments for patients with normal to mild cognitive impairment via telerehabilitation | | | E. Ward et al. | 2012 |
|  | 6 | | |  | | Teleneuropsychology: Evidence for video teleconference-based neuropsychological assessment | | | C. Cullum et al. | 2014 |
|  | 7 | | |  | | Efficacy of Technology-delivered Cognitive Behavioural Therapy for OCD Versus Control Conditions, and in Comparison with Therapist-Administered CBT: Meta-Analysis of Randomized Controlled Trials | | | D. Dettore et al. | 2015 |
|  | 8 | | |  | | Telemedicine for children with developmental disabilities: a more effective clinical process than office-based care | | | D. Langkamp et al. | 2015 |
|  | 9 | | |  | | Guided Internet-based versus face-to-face clinical care in the management of tinnitus: Study protocol for a multi-centre randomised controlled trial | | | E. W. Beukes et al. | 2017 |
|  | 10 | | |  | | Investigating the accuracy of a novel telehealth diagnostic approach for autism spectrum disorder | | | C. Smith et al. | 2017 |
|  | 11 | | |  | | A new device-aided cognitive function test, User eXperience-Trail Making Test (UX-TMT), sensitively detects neuropsychological performance in patients with … | | | N. Kokubo et al. | 2018 |
|  | 12 | | |  | | Pragmatic RAndomised controlled trial of a trauma-focused guided self-help Programme versus InDividual traumafocused cognitive Behavioural therapy for post-traumatic stress disorder (RAPID): Trial protocol | | | C. Nollett et al. | 2018 |
|  | 13 | | |  | | Limitations of integrating technology in cognitive testing of non-verbal memory | | | A. Porrselvi and V. Shankar | 2018 |
|  | 14 | | |  | | Study protocol for a single-blind, randomised controlled, non-inferiority trial of internet-based versus face-to-face cognitive behaviour therapy for obsessive-compulsive disorder | | | C. Ruck et al. | 2018 |
|  | 15 | | |  | | Improving the efficiency of psychotherapy for depression: Computer-assisted versus standard CBT | | | M. E. Thase et al. | 2018 |
|  | 16 | | |  | | Agreement between telehealth and face-to-face assessment of intellectual ability in children with specific learning disorder | | | M. Hodge et al. | 2019 |
|  | 17 | | |  | | The Internet Intervention Patient Adherence Scale for Guided Internet-Delivered Behavioral Interventions: Development and Psychometric Evaluation | | | F. Lenhard et al. | 2019 |
|  | 18 | | |  | | The Oxford Cognitive Screen–Plus (OCS-Plus): A digital, tablet-based, brief cognitive assessment | | | N. Demeyere et al. | 2020 |
|  | 19 | | |  | | Home-based pediatric teleneuropsychology: A validation study | | | L. Harder et al. | 2020 |
|  | 20 | | |  | | Therapeutic Relationship in eHealth-A Pilot Study of Similarities and Differences between the Online Program Priovi and Therapists Treating Borderline Personality Disorder | | | S. Köhne et al. | 2020 |
|  | 21 | | |  | | Taking language samples home: Feasibility, reliability, and validity of child language samples conducted remotely with video chat versus in-person | | | B. Manning et al. | 2020 |
|  | 22 | | |  | | Behavioral couples therapy versus cognitive behavioral therapy for problem gambling: A randomized controlled trial | | | A. Nilsson et al. | 2020 |
|  | 23 | | |  | | Reliability and validity of a novel caregiver-assessed skills system based on the ALSO conception in children with autism spectrum disorders | | | Y. Chen et al. | 2021 |
|  | 24 | | |  | | Q-interactive: Training Implications for Accuracy and Technology Integration | | | S. Corcoran | 2021 |
|  | 25 | | |  | | The Brief Observation of Symptoms of Autism (BOSA): Development of a New Adapted Assessment Measure for Remote Telehealth Administration through … | | | D. Dow et al. | 2021 |
|  | 26 | | |  | | Psychotic experiences in daily-life in adolescents and young adults with 22q11. 2 deletion syndrome: An Ecological Momentary Assessment study | | | C. Feller et al. | 2021 |
|  | 27 | | |  | | Balancing access with technology: comparing in-person and telerehabilitation Berg Balance Scale scores among stroke survivors | | | D. Gillespie et al. | 2021 |
|  | 28 | | |  | | The Boston cognitive assessment: Psychometric foundations of a self-administered measure of global cognition | | | D. Gold et al. | 2021 |
|  | 29 | | |  | | Equivalency of in-person versus remote assessment: WISC-V and KTEA-3 performance in clinically referred children and adolescents | | | T. Hamner et al. | 2021 |
|  | 30 | | |  | | Internet-based therapy versus face-to-face therapy for alcohol use disorder, a randomized controlled non-inferiority trial | | | M. Johansson et al. | 2021 |
|  | 31 | | |  | | Effect of Game-Based Cognitive Training Programs on Cognitive Learning of Children with Intellectual Disabilities | | | S. Kim and H. Lee | 2021 |
|  | 32 | | |  | | Remote assessment of verbal memory in youth with cochlear implants during the COVID-19 pandemic | | | W. Kronenberger et al. | 2021 |
|  | 33 | | |  | | Remote Assessment of Depression Using Digital Biomarkers From Cognitive Tasks | | | R. Mandryk et al. | 2021 |
|  | 34 | | |  | | Zoom, Zoom, Baby! Assessing Mother-Infant Interaction During the Still Face Paradigm and Infant Language Development via a Virtual Visit Procedure | | | N. McElwain et al. | 2021 |
|  | 35 | | |  | | Feasibility and initial validation of 'HD-Mobile', a smartphone application for remote self-administration of performance-based cognitive measures in Huntington's … | | | B. McLaren et al. | 2021 |
|  | 36 | | |  | | Feasibility of remote assessment of the binaural intelligibility level difference in school-age children | | | G. Merchant et al. | 2021 |
|  | 37 | | |  | | Comparing Face-to-Face and Online Data Collection Methods in Preterm and Full-Term Children: An Exploratory Study | | | P. Nelson et al. | 2021 |
|  | 38 | | |  | | Feasibility of Remote Performance Assessment Using the Free Research Executive Evaluation Test Battery in Adolescents | | | I. Segura and S. Pompéia | 2021 |
|  | 39 | | |  | | The V-5 provides quick, accurate and cross-culturally valid measures of psychiatric symptoms | | | C. Sirianni et al. | 2021 |
|  | 40 | | |  | | The digital marshmallow test (DMT) diagnostic and monitoring Mobile health app for impulsive behavior: development and validation study | | | M. Sobolev et al. | 2021 |
|  | 41 | | |  | | Research at a distance: replicating semantic differentiation effects using remote data collection with children participants | | | C. Vales et al. | 2021 |
|  | 42 | | |  | | Can the Rorschach be Administered Remotely? A Review of Options and a Pilot Study Using a Newly Developed R-PAS App | | | F. Ales et al. | 2022 |
|  | 43 | | |  | | FarmApp: a new assessment of cognitive control and memory for children and young people with neurodevelopmental difficulties | | | D. Brkić et al. | 2022 |
|  | 44 | | |  | | Online Administration of the Test of Narrative Language–Second Edition: Psychometrics and Considerations for Remote Assessment | | | B. Magimairaj et al. | 2022 |
|  | 45 | | |  | | From a distance: Comparison of in-person and virtual assessments with adult–child dyads from linguistically diverse backgrounds | | | A. Pratt et al. | 2022 |
|  |  | | |  | | ***Guides/Protocols/Instruments*** | | |  |  |
|  | 1 | | |  | | A portable telerehabilitation system for remote evaluations of impaired elbows in neurological disorders | | | H. Park et al. | 2008 |
|  | 2 | | |  | | Design, development, and evaluation of an online virtual emergency department for training trauma teams | | | P. Youngblood et al. | 2008 |
|  | 3 | | |  | | A Roadmap to Computer-Based Psychotherapy in the United States | | | J. A. Cartreine et al. | 2010 |
|  | 4 | | |  | | Content and functionality of alcohol and other drug websites: Results of an online survey | | | B. Klein et al. | 2010 |
|  | 5 | | |  | | Evidence-based practice for telemental health | | | B. Grady et al. | 2011 |
|  | 6 | | |  | | A web-based normative calculator for the uniform data set (UDS) neuropsychological test battery | | | S. D. Shirk et al. | 2011 |
|  | 7 | | |  | | Transdiagnostic, affect-focused, psychodynamic, guided self-help for depression and anxiety through the internet: study protocol for a randomised controlled trial | | | R. Johansson et al. | 2012 |
|  | 8 | | |  | | Behavioral activation-based guided self-help treatment administered through a smartphone application: Study protocol for a randomized controlled trial | | | K. H. Ly et al. | 2012 |
|  | 9 | | |  | | Design of a Web-based individual coping and alcohol-intervention program (web-ICAIP) for children of parents with alcohol problems: study protocol for a randomized controlled trial | | | T. H. Elgan et al. | 2012 |
|  | 10 | | |  | | Behavioral activation-based guided self-help treatment administered through a smartphone application: Study protocol for a randomized controlled trial | | | K. H. Ly, P. Carlbring and G. Andersson | 2012 |
|  | 11 | | |  | | Bi-factor analyses of the Brief Test of Adult Cognition by Telephone | | | B. Gavett et al. | 2013 |
|  | 12 | | |  | | Using the internet to provide psychodynamic psychotherapy | | | R. Johansson et al. | 2013 |
|  | 13 | | |  | | Development and initial evaluation of an internet-based support system for face-to-face cognitive behavior therapy: A proof of concept study | | | K. N. T. Månsson et al. | 2013 |
|  | 14 | | |  | | Development and Usability Testing of an Internet Intervention to Increase Physical Activity in Overweight Adolescents | | | K. Riiser et al. | 2013 |
|  | 15 | | |  | | Bi-factor analyses of the Brief Test of Adult Cognition by Telephone | | | B. Gavett et al. | 2013 |
|  | 16 | | |  | | Web-Based Cognitive Behavioral Relapse Prevention Program With Tailored Feedback for People With Methamphetamine and Other Drug Use Problems: Development and Usability Study | | | A. Takanol et al. | 2016 |
|  | 17 | | |  | | Internet-delivered cognitive therapy for PTSD: a development pilot series | | | J. Wild et al. | 2016 |
|  | 18 | | |  | | A bespoke mobile application for the longitudinal assessment of depression and mood during pregnancy: protocol of a feasibility study | | | J. Belisario et al. | 2017 |
|  | 19 | | |  | | Internet-delivered cognitive behavioral therapy for posttraumatic stress disorder in international humanitarian aid workers: Study protocol | | | I. Kunovski et al. | 2017 |
|  | 20 | | |  | | Investigating the accuracy of a novel telehealth diagnostic approach for autism spectrum disorder | | | C. Smith et al. | 2017 |
|  | 21 | | |  | | Pragmatic RAndomised controlled trial of a trauma-focused guided self-help Programme versus InDividual traumafocused cognitive Behavioural therapy for post-traumatic stress disorder (RAPID): Trial protocol | | | C. Nollett et al. | 2018 |
|  | 22 | | |  | | Development of an internet-administered cognitive behavior therapy program (ENGAGE) for parents of children previously treated for cancer: Participatory action research approach | | | A. Wikman et al. | 2018 |
|  | 23 | | |  | | Study protocol for a single-blind, randomised controlled, non-inferiority trial of internet-based versus face-to-face cognitive behaviour therapy for obsessive-compulsive disorder | | | C. Ruck et al. | 2018 |
|  | 24 | | |  | | Investigating a therapist-guided, parent-assisted remote digital behavioural intervention for tics in children and adolescents-'Online Remote Behavioural Intervention for Tics' (ORBIT) trial: protocol of an internal pilot study and single-blind randomised controlled trial | | | C. L. Hall et al. | 2019 |
|  | 25 | | |  | | Remote assessment of cognitive impairment level based on serious mobile game performance: an initial proof of concept | | | H. Jung et al. | 2019 |
|  | 26 | | |  | | Using internet-based self-help to bridge waiting time for face-to-face outpatient treatment for Bulimia Nervosa, Binge Eating Disorder and related disorders: Study protocol of a randomized controlled trial | | | B. Vollert et al. | 2019 |
|  | 27 | | |  | | Analysis, Design, and Prototypical Implementation of a Serious Game Reha@Stroke to Support Rehabilitation of Stroke Patients With the Help of a Mobile Phone | | | R. Baranyi et al. | 2020 |
|  | 28 | | |  | | InterOrganizational practice committee recommendations/guidance for teleneuropsychology (TeleNP) in response to the COVID-19 pandemic | | | R. Bilder et al. | 2020 |
|  | 29 | | |  | | Moving toward telehealth surveillance services for toddlers at risk for autism during the COVID-19 pandemic | | | E. Conti et al. | 2020 |
|  | 30 | | |  | | Teleassessment with Children and Adolescents During the Coronavirus (COVID-19) Pandemic and | | | R. Farmer et al. | 2020 |
|  | 31 | | |  | | Emory university telehealth neuropsychology development and implementation in response to the COVID-19 pandemic | | | K. Hewitt and D. Loring | 2020 |
|  | 32 | | |  | | The COVID-19 pandemic and treating suicidal risk: The telepsychotherapy use of CAMS | | | D. Jobes et al. | 2020 |
|  | 33 | | |  | | Remote testing for psychological and physiological acoustics: Initial report of the p&p task force on remote testing | | | Z. Peng et al. | 2020 |
|  | 34 | | |  | | Pediatric teleneuropsychology: Feasibility and recommendations | | | D. Ransom et al. | 2020 |
|  | 35 | | |  | | Treating posttraumatic stress disorder remotely with cognitive therapy for PTSD | | | J. Wild et al. | 2020 |
|  | 36 | | |  | | Analysis, Design, and Prototypical Implementation of a Serious Game Reha@Stroke to Support Rehabilitation of Stroke Patients With the Help of a Mobile Phone | | | R. Baranyi et al. | 2020 |
|  | 37 | | |  | | InterOrganizational practice committee recommendations/guidance for teleneuropsychology (TeleNP) in response to the COVID-19 pandemic | | | R. Bilder et al. | 2020 |
|  | 38 | | |  | | BrainTracker: mHealth app for Remote Assessment of Pediatric Epilepsy and Comorbidities | | | K. Gary et al. | 2020 |
|  | 39 | | |  | | Emory university telehealth neuropsychology development and implementation in response to the COVID-19 pandemic | | | K. Hewitt and D. Loring | 2020 |
|  | 40 | | |  | | Leveraging technology to improve military mental health: Novel uses of smartphone apps | | | E. Vermetten et al. | 2020 |
|  | 41 | | |  | | Feasibility, acceptability, and effectiveness of enhanced cognitive behavioral therapy (eCBT) for children and adolescents with obsessive-compulsive disorder: Protocol for an open trial and therapeutic intervention | | | L. H. Wolters et al. | 2020 |
|  | 42 | | |  | | From Lab to Zoom: Adapting Training Study Methodologies to Remote Conditions | | | V. P. Bambha and M. Casasola | 2021 |
|  | 43 | | |  | | Working Group Recommendations for the Practice of Teleneuropsychology in Latin America | | | L. Crivelli et al. | 2021 |
|  | 44 | | |  | | Early Moves: a protocol for a population-based prospective cohort study to establish general movements as an early biomarker of cognitive impairment in infants | | | C. Elliott et al. | 2021 |
|  | 45 | | |  | | Innovative methods for remote assessment of neurobehavioral development | | | H. Gustafsson et al. | 2021 |
|  | 46 | | |  | | ImaginYouth—A Therapist-Guided Internet-Based Cognitive-Behavioral Program for Adolescents and Young Adults With Body Dysmorphic Disorder: Study Protocol for a Two-Arm Randomized Controlled Trial | | | A. S. Hartmann et al. | 2021 |
|  | 47 | | |  | | Disruption Leads to Methodological and Analytic Innovation in Developmental Sciences: Recommendations for Remote Administration and Dealing With … | | | S. Krogh-Jespersen et al. | 2021 |
|  | 48 | | |  | | Teleneuropsychology: A model for clinical practice | | | L. Thibodaux et al. | 2021 |
|  | 49 | | |  | | Development and evaluation of an internet-based cognitive behavioral therapy intervention for anxiety and depression in adults with cystic fibrosis (eHealth CF-CBT): An international collaboration | | | M. Verkleij et al. | 2021 |
|  | 50 | | |  | | Internet-administered cognitive behavioral therapy for common mental health difficulties in parents of children treated for cancer: Intervention development and description study | | | J. Woodford et al. | 2021 |
|  | 51 | | |  | | ImaginYouth—A Therapist-Guided Internet-Based Cognitive-Behavioral Program for Adolescents and Young Adults With Body Dysmorphic Disorder: Study Protocol for a Two-Arm Randomized Controlled Trial | | | A. S. Hartmann et al. | 2021 |
|  | 52 | | |  | | Integrating internet CBT into clinical practice: a practical guide for clinicians | | | J. Newby et al. | 2021 |
|  | 53 | | |  | | A case series illustrating the implementation of a novel tele-neuropsychology service model during COVID-19 for children with complex medical and … | | | R. Peterson et al. | 2021 |
|  | 54 | | |  | | Teleneuropsychology: A model for clinical practice | | | L. Thibodaux et al. | 2021 |
|  | 55 | | |  | | A virtual reality-based cognitive telerehabilitation system for use in the COVID-19 pandemic | | | J. Varela-Aldás et al. | 2021 |
|  | 56 | | |  | | Internet-administered cognitive behavioral therapy for common mental health difficulties in parents of children treated for cancer: Intervention development and description study | | | J. Woodford et al. | 2021 |
|  | 57 | | |  | | Best Practice During Teleconsultations With Adolescents: A Scoping Review | | | S. Davidson et al. | 2022 |
|  | ***Technical/Usability*** | | | | | | | | | |
|  | 1 | | |  | | Intelligent technology for an aging population: The use of AI to assist elders with cognitive impairment | | | M. Pollack | 2005 |
|  | 2 | | |  | | Tracer: A general-purpose software library for logging events in computerized experiments | | | O. Lahl and R. Pietrowsky | 2008 |
|  | 3 | | |  | | The use of telephone speech recordings for assessment and monitoring of cognitive function in elderly people | | | V. Rapcan et al. | 2009 |
|  | 4 | | |  | | Consumer acceptability of brief videoconference-based neuropsychological assessment in older individuals with and without cognitive impairment | | | M. Parikh et al. | 2013 |
|  | 5 | | |  | | An integrated telehealth system for remote administration of an adult autism assessment | | | B. Parmanto et al. | 2013 |
|  | 6 | | |  | | Telehealth technology applications in speech-language pathology | | | C. Keck and C. Doarn | 2014 |
|  | 7 | | |  | | SIG-Blocks: Tangible game technology for automated cognitive assessment | | | K. Lee et al. | 2016 |
|  | 8 | | |  | | Bonn eXperimental System (BoXS): An open-source platform for interactive experiments in psychology and economics | | | M. Seithe et al. | 2016 |
|  | 9 | | |  | | Web-Based Cognitive Behavioral Relapse Prevention Program With Tailored Feedback for People With Methamphetamine and Other Drug Use Problems: Development and Usability Study | | | A. Takanol et al. | 2016 |
|  | 10 | | |  | | How to build a supervised autonomous system for robot-enhanced therapy for children with autism spectrum disorder | | | P. G. Esteban et al. | 2017 |
|  | 11 | | |  | | Tips and traps: lessons from codesigning a clinician e-monitoring tool for computerized cognitive behavioral therapy | | | F. Sundram et al. | 2017 |
|  | 12 | | |  | | Objective User Engagement With Mental Health Apps: Systematic Search and Panel-Based Usage Analysis | | | A. Baumel et al. | 2019 |
|  | 13 | | |  | | A thousand studies for the price of one: Accelerating psychological science with Pushkin | | | J. K. Hartshorne et al. | 2019 |
|  | 14 | | |  | | Remote assessment of cognitive impairment level based on serious mobile game performance: an initial proof of concept | | | H. Jung et al. | 2019 |
|  | 15 | | |  | | SMARTRIQS: A Simple Method Allowing Real-Time Respondent Interaction in Qualtrics Surveys | | | A. Molnar | 2019 |
|  | 16 | | |  | | Engaging across dimensions of diversity: a cross-national perspective on mHealth tools for managing relapsing remitting and progressive multiple sclerosis | | | S. Simblett et al. | 2019 |
|  | 17 | | |  | | Analysis, Design, and Prototypical Implementation of a Serious Game Reha@Stroke to Support Rehabilitation of Stroke Patients With the Help of a Mobile Phone | | | R. Baranyi et al. | 2020 |
|  | 18 | | |  | | BrainTracker: mHealth app for Remote Assessment of Pediatric Epilepsy and Comorbidities | | | K. Gary et al. | 2020 |
|  | 19 | | |  | | Bringing the laboratory home: PANDABox telehealth-based assessment of neurodevelopmental risk in children | | | B. Kelleher et al. | 2020 |
|  | 20 | | |  | | Usability evaluation of a tangible user interface and serious game for identification of cognitive deficiencies in preschool children | | | A. Sánchez-Morales et al. | 2020 |
|  | 21 | | |  | | A simple and effective way to study executive functions by using 360 videos | | | F. Borgnis et al. | 2021 |
|  | 22 | | |  | | Identifying Mild Cognitive Impairment by Using Human–Robot Interactions | | | Y. Chang et al. | 2021 |
|  | 23 | | |  | | Social robots for evaluating attention state in older adults | | | Y. Chen et al. | 2021 |
|  | 24 | | |  | | Humanoid Robot Based Platform to Evaluate the Efficacy of Using Inertial Sensors for Spasticity Assessment in Cerebral Palsy | | | N. Cooney and A. Minhas | 2021 |
|  | 25 | | |  | | Assessing social anxiety through digital biomarkers embedded in a gaming task | | | M. J. Dechant et al. | 2021 |
|  | 26 | | |  | | Evaluation of a synthetic version of the digits-in-noise test and its characteristics in CI recipients | | | M. Kropp et al. | 2021 |
|  | 27 | | |  | | Bayesian Mixed Effects Model and Data Visualization for Understanding Item Response Time and Response Order in Open Online Assessment | | | Y. Liu et al. | 2021 |
|  | 28 | | |  | | Decoding depressive disorder using computer vision | | | J. Singh and G. Goyal | 2021 |
|  | 29 | | |  | | A virtual reality-based cognitive telerehabilitation system for use in the COVID-19 pandemic | | | J. Varela-Aldás et al. | 2021 |
|  | 30 | | |  | | HOPES: an integrative digital phenotyping platform for data collection, monitoring, and machine learning | | | X. Wang et al. | 2021 |
|  | 31 | | |  | | Remote and at-home data collection: Considerations for the NIH HEALthy Brain and Cognitive Development (HBCD) study | | | S. Deoni et al. | 2022 |
|  | 32 | | |  | | Robotics Applications for Public Health and Safety During the COVID-19 Pandemic | | | M. Javaid et al. | 2022 |
|  | 33 | | |  | | The Development of an mHealth Tool for Children With Long-term Illness to Enable Person-Centered Communication: User-Centered Design Approach | | | A. Wiljén et al. | 2022 |
|  | ***Tele-Education*** | | | | | | | | | |
|  | 1 | | |  | | Toward the development of analysis of students' cognitive processes in an online course | | | R. S. Shieh | 2006 |
|  | 2 | | |  | | Videoconferencing and telehealth technologies can provide a reliable approach to remote assessment and teaching without compromising quality | | | J. Winters et al. | 2007 |
|  | 3 | | |  | | Assessment of children's literacy via an Internet-based telehealth system | | | M. Waite et al. | 2010 |
|  | 4 | | |  | | Testing practices in the 21st century | | | A. Evers et al. | 2012 |
|  | 5 | | |  | | Exploring students' cognitive dimensions and behavioral patterns during a synchronous peer assessment discussion activity using instant messaging | | | S. Y. Wu et al. | 2012 |
|  | 6 | | |  | | Collab-Analyzer: An environment for conducting web-based collaborative learning activities and analyzing students' information-searching behaviors | | | C. H. Wu et al. | 2014 |
|  | 7 | | |  | | Distance Learning Approaches in the Mathematical Training of Pedagogical Institutes's Students | | | T. Fomina et al. | 2016 |
|  | 8 | | |  | | What 'World of Warcraft' is teaching us about learning | | | L. K. Ackerman | 2018 |
|  | 9 | | |  | | Promoting self-paced learning in the elementary classroom with interactive video, an online course platform and tablets | | | G. Palaigeorgiou and A. Papadopoulou | 2019 |
|  | 10 | | |  | | Investigating the Transition to Remote Teaching During COVID-19 | | | A. Aebersold et al. | 2020 |
|  | 11 | | |  | | School&Apos; S Out: Experimental Evidence on Limiting Learning Loss Using | | | N. Angrist et al. | 2020 |
|  | 12 | | |  | | Testing our children when the world shuts down: Analyzing recommendations for adapted tele-assessment during COVID-19 | | | S. Krach et al. | 2020 |
|  | 13 | | |  | | Remote Assessing Children's Handwriting Spelling on Mobile Devices | | | J. Mombach et al. | 2020 |
|  | 14 | | |  | | Transforming Assessment in Response to COVID-19 | | | T. Dhurumraj et al. | 2021 |
|  | 15 | | |  | | Examining effectiveness of online teaching modules on Developmentally Appropriate Practices (DAP) for guiding young children's behavior: student and instructor perspectives | | | A. V. Hegde and B. S. Hewett | 2021 |
|  | 16 | | |  | | Examining Ease and Challenges in Tele-Assessment of Children Using Slosson Intelligence Test | | | R. Jaffar and A. Ali | 2021 |
|  | 17 | | |  | | Effect of Game-Based Cognitive Training Programs on Cognitive Learning of Children with Intellectual Disabilities | | | S. Kim and H. Lee | 2021 |
|  | 18 | | |  | | Remote assessment of verbal memory in youth with cochlear implants during the COVID-19 pandemic | | | W. Kronenberger et al. | 2021 |
|  | 19 | | |  | | The short-term impact of remote instruction on achievement in children with ADHD during the COVID-19 pandemic | | | K. Lupas et al. | 2021 |
|  | 20 | | |  | | Research trends in online distance learning during the COVID-19 pandemic | | | S. Mishra et al. | 2021 |
|  | 21 | | |  | | Problems of students' assessment in the process of distance learning (on the example of the EU countries) | | | N. Ridei et al. | 2021 |
|  | 22 | | |  | | Shaping open, distance and e-learning in post school education and training: A call for a revised agenda | | | R. Aluko et al. | 2022 |
|  | 23 | | |  | | Tele-Assessments in Rural and Remote Schools–Perspectives of Support Teachers | | | M. Hodge et al. | 2022 |
|  | 24 | | |  | | The effects of virtual assessment on capturing skill growth in children with hearing loss | | | E. Lund and K. Werfel | 2022 |
|  | 25 | | |  | | Evaluating the feasibility of remotely administered curriculum‐based measurement for students with autism: A pilot study | | | S. Mire et al. | 2022 |
|  | 26 | | |  | | Assessment for an Unprecedented Education | | | T. Victorița | 2022 |
|  | 27 | | |  | | Using Technology to Assess Individualized Education Plan Goal Progress During Virtual Learning | | | T. Wade et al. | 2022 |
|  | ***Ethics/data management*** | | | | | | | | | |
| 1 | |  |  | | | | Neuroethical and societal challenges of 21st century epidemics | | N. Minielly et al. | 2020 |
| 2 | |  |  | | | | Assessment during the COVID-19 pandemic: Ethical, legal, and safety considerations moving forward | | S. Stifel et al. | 2020 |
| 3 | |  |  | | | | Using Security Questions to Link Participants in Longitudinal Data Collection | | S. Xu, A et al. | 2020 |
| 4 | |  |  | | | | Limits of remote working: the ethical challenges in conducting Mental Health Act assessments during COVID-19 | | L. Schölin et al. | 2021 |
| 5 | |  |  | | | | Standards for Objectivity and Reproducibility in High-Impact Developmental Studies—The COVID-19 Pandemic and Beyond | | M. Thomason | 2021 |
|  | ***Theoretical/Reviews/Meta-analyses*** | | | | | | | | | |
|  | 1 | | |  | | | Maria Devita, Alessandra Bordignon | | G. Sergi and A. Coin | |
|  | 2 | | |  | | | Telerehabilitation research: emerging opportunities | | J. Winters | 2002 |
|  | 3 | | |  | | | Outcomes of an integrated telehealth network demonstration project | | S. Dimmick et al. | 2003 |
|  | 4 | | |  | | | Telemedicine in the state of Maine: A model for growth driven by rural needs | | M. Edwards and A. Patel | 2003 |
|  | 5 | | |  | | | Telemedicine and neurosciences | | K. Ganapathy | 2005 |
|  | 6 | | |  | | | Can telepsychiatry replace in-person psychiatric assessments? A review and meta-analysis of comparison studies | | S. Hyler et al. | 2005 |
|  | 7 | | |  | | | Intelligent technology for an aging population: The use of AI to assist elders with cognitive impairment | | M. Pollack | 2005 |
|  | 8 | | |  | | | Retrieving e-health research: The challenge of accessing the knowledge | | R. Davis et al. | 2006 |
|  | 9 | | |  | | | The application of telemedicine to geriatric medicine | | M. Brignell et al. | 2007 |
|  | 10 | | |  | | | Telepsychiatry assessments of child or adolescent behavior disorders: a review of evidence and issues | | J. Diamond and R. Bloch | 2010 |
|  | 11 | | |  | | | e-Health and chronic pain management: current status and developments | | E. Keogh et al. | 2010 |
|  | 12 | | |  | | | Enhancing quality of life through telerehabilitation | | M. McCue et al. | 2010 |
|  | 13 | | |  | | | Ten years of telerehabilitation: A literature overview of technologies and clinical applications | | M. Rogante et al. | 2010 |
|  | 14 | | |  | | | Evidence-based practice for telemental health | | B. Grady et al. | 2011 |
|  | 15 | | |  | | | Clinical telerehabilitation: applications for physiatrists | | P. Gregory et al. | 2011 |
|  | 16 | | |  | | | Traumatic brain injury | | J. Risdall and D. Menon | 2011 |
|  | 17 | | |  | | | The use of videoconferencing with patients with psychosis: a review of the literature | | I. Sharp et al. | 2011 |
|  | 18 | | |  | | | Internet-Based Approaches to Collaborative Therapeutic Assessment: New Opportunities for Professional Psychologists | | R. E. Smith et al. | 2011 |
|  | 19 | | |  | | | Therapeutic videoconferencing interventions for the treatment of long-term conditions | | K. Steel et al. | 2011 |
|  | 20 | | |  | | | Telepsychiatry and e-Mental Health | | A. Fishkind et al. | 2012 |
|  | 21 | | |  | | | A new era in speech-language pathology practice: Innovation and diversification | | D. Theodoros | 2012 |
|  | 22 | | |  | | | Challenges and opportunities in internet-mediated telemental health | | E. Yuen et al. | 2012 |
|  | 23 | | |  | | | Telehealth | | D. J. Cason et al. | 2013 |
|  | 24 | | |  | | | Facilitating stroke management using modern information technology | | H. Nam et al. | 2013 |
|  | 25 | | |  | | | Tele-health and neurology: what is possible? | | F. Timpano et al. | 2013 |
|  | 26 | | |  | | | Teleneuropsychology: Evidence for video teleconference-based neuropsychological assessment | | C. Cullum et al. | 2014 |
|  | 27 | | |  | | | Telehealth technology applications in speech-language pathology | | C. Keck and C. Doarn | 2014 |
|  | 28 | | |  | | | Opportunities and challenges of internet-based health interventions in the future internet | | A. Lim and C. Thuemmler | 2015 |
|  | 29 | | |  | | | A systematic review of the use of telehealth in speech, language and hearing sciences | | D. R. Molini-Avejonas et al. | 2015 |
|  | 30 | | |  | | | State of telehealth | | E. Dorsey and E. Topol | 2016 |
|  | 31 | | |  | | | Videoconferencing in psychiatry, a meta-analysis of assessment and treatment | | A. Drago et al. | 2016 |
|  | 32 | | |  | | | Review of key telepsychiatry outcomes | | S. Hubley et al. | 2016 |
|  | 33 | | |  | | | Internet interventions for depression: new developments | | J. Schroder et al. | 2016 |
|  | 34 | | |  | | | Overcoming barriers to using telehealth for standardized language assessments | | R. Sutherland et al. | 2016 |
|  | 35 | | |  | | | Three decades of telemedicine in obsessive-compulsive disorder: a review across platforms | | E. Aboujaoude | 2017 |
|  | 36 | | |  | | | Annual Research Review: Digital health interventions for children and young people with mental health problems–a systematic and meta‐review | | C. Hollis et al. | 2017 |
|  | 37 | | |  | | | Telehealth use in Speech-Language Pathology: An exploratory scoping review | | L. Krikheli et al. | 2017 |
|  | 38 | | |  | | | The current state of telehealth evidence: a rapid review | | E. Shigekawa et al. | 2018 |
|  | 39 | | |  | | | Use of nonintrusive sensor-based information and communication technology for real-world evidence for clinical trials in dementia | | S. Teipel et al. | 2018 |
|  | 40 | | |  | | | eHealth interventions for anxiety management targeting young children and adolescents: Exploratory review | | F. Tozzi et al. | 2018 |
|  | 41 | | |  | | | Internet‐delivered psychological treatments: From innovation to implementation | | G. Andersson et al. | 2019 |
|  | 42 | | |  | | | Is there a trial bias impacting user engagement with unguided e-mental health interventions? A systematic comparison of published reports and real-world usage of … | | A. Baumel et al. | 2019 |
|  | 43 | | |  | | | Telemedicine in rehabilitation | | M. Galea | 2019 |
|  | 44 | | |  | | | Review on psychological stress detection using biosignals | | G. Giannakakis et al. | 2019 |
|  | 45 | | |  | | | Use of telepsychiatry in emergency and crisis intervention: current evidence | | I. Reinhardt et al. | 2019 |
|  | 46 | | |  | | | A New AAN Report Details Where There Is Evidence for Teleneurology—And Where There Are Gaps | | G. Shaw | 2019 |
|  | 47 | | |  | | | Pediatric telehealth: Approaches by specialty and implications for general pediatric care | | A. Tomines | 2019 |
|  | 48 | | |  | | | A systematic review into the assessment of medical apps: motivations, challenges, recommendations and methodological aspect | | A. Alamoodi et al. | 2020 |
|  | 49 | | |  | | | Adherence to established treatment guidelines among unguided digital interventions for depression: Quality evaluation of 28 web-based programs and mobile apps | | S. Bubolz et al. | 2020 |
|  | 50 | | |  | | | A systematic review of remote telehealth assessments for early signs of autism spectrum disorder: Video and mobile applications | | A. Dahiya et al. | 2020 |
|  | 51 | | |  | | | Telemedicine in neurology: current evidence | | R. Domingues et al. | 2020 |
|  | 52 | | |  | | | Emory university telehealth neuropsychology development and implementation in response to the COVID-19 pandemic | | K. Hewitt and D. Loring | 2020 |
|  | 53 | | |  | | | Transitioning to telehealth neuropsychology service: Considerations across adult and pediatric care settings | | K. Hewitt et al. | 2020 |
|  | 54 | | |  | | | The COVID-19 pandemic and treating suicidal risk: The telepsychotherapy use of CAMS | | D. Jobes et al. | 2020 |
|  | 55 | | |  | | | Introduction to Special Issue on Outcome Measures for IDD: Where We Have Been, Where We Are Now, and Where We Are Heading | | B. Kelleher and A. Wheeler | 2020 |
|  | 56 | | |  | | | Neuroethical and societal challenges of 21st century epidemics | | N. Minielly et al. | 2020 |
|  | 57 | | |  | | | Pediatric teleneuropsychology: Feasibility and recommendations | | D. Ransom et al. | 2020 |
|  | 58 | | |  | | | Teleneuropsychology in the time of COVID-19: the experience of The Australian Epilepsy Project | | C. Tailby et al. | 2020 |
|  | 59 | | |  | | | Telepsychiatry and outpatient department services | | L. Vadlamani et al. | 2020 |
|  | 60 | | |  | | | A systematic review evaluating the implementation of technologies to assess, monitor and treat neurodevelopmental disorders: A map of the current evidence | | A. Valentine et al. | 2020 |
|  | 61 | | |  | | | Internet-delivered cognitive behavioral therapies for late-life depressive symptoms: A systematic review and meta-analysis | | X. Xiang et al. | 2020 |
|  | 62 | | |  | | | Assessment of Psychological Distress in Adults With Type 2 Diabetes Mellitus Through Technologies: Literature Review | | G. Bassi et al. | 2021 |
|  | 63 | | |  | | | Are videoconferenced mental and behavioral health services just as good as in-person? A meta-analysis of a fast-growing practice | | A. Batastini et al. | 2021 |
|  | 64 | | |  | | | A Perspective on Client-Psychologist Relationships in Videoconferencing Psychotherapy: Literature Review | | F. Cataldo et al. | 2021 |
|  | 65 | | |  | | | Acceptance, adoption, and usability of information and communication technologies for people living with dementia and their care partners: A systematic review | | A. M. Cruz et al. | 2021 |
|  | 66 | | |  | | | Real-time mobile monitoring of bipolar disorder: a review of evidence and future directions | | G. Dunster et al. | 2021 |
|  | 67 | | |  | | | Telehealth and autism prior to and in the age of COVID-19: a systematic and critical review of the last decade | | K. Ellison et al. | 2021 |
|  | 68 | | |  | | | School-based suicide risk assessment using ehealth for youth: systematic scoping review | | D. Exner-Cortens et al. | 2021 |
|  | 69 | | |  | | | Clinical psychological assessment training issues in the COVID-19 era: A survey of the state of the field and considerations for moving forward | | K. Gicas, T. Paterson et al. | 2021 |
|  | 70 | | |  | | | Pediatric neurology and telehealth before and during SARS-CoV-2 pandemic | | B. Golebiowska and M. Golebiowska | 2021 |
|  | 71 | | |  | | | Emerging Needs and Viability of Telepsychiatry During and Post COVID-19 Era: A Literature Review | | J. Gude et al. | 2021 |
|  | 72 | | |  | | | Tele-NeuroRehabilitation | | A. Hill et al. | 2021 |
|  | 73 | | |  | | | Telehealth in pediatric rehabilitation | | N. Hsu et al. | 2021 |
|  | 74 | | |  | | | Integrating internet CBT into clinical practice: a practical guide for clinicians | | J. Newby et al. | 2021 |
|  | 75 | | |  | | | Information and communication technology use in suicide prevention: Scoping Review | | J. Rassy et al. | 2021 |
|  | 76 | | |  | | | Virtual health care for adult patients with intellectual and developmental disabilities: A scoping review | | A. Selick et al. | 2021 |
|  | 77 | | |  | | | Teleneuropsychological Assessment During the COVID-19 Pandemic: Where do we go from here? | | M. Sharma et al. | 2021 |
|  | 78 | | |  | | | Wearable, environmental, and smartphone-based passive sensing for mental health monitoring | | M. Sheikh et al. | 2021 |
|  | 79 | | |  | | | Digital Technology for Remote Hearing Assessment—Current Status and Future Directions for Consumers | | K. D. Sousa et al. | 2021 |
|  | 80 | | |  | | | Virtual reality and wearable technologies to support adaptive responding of children and adolescents with neurodevelopmental disorders: a critical comment … | | F. Stasolla | 2021 |
|  | 81 | | |  | | | Teleneuropsychology: A model for clinical practice | | L. Thibodaux et al. | 2021 |
|  | 82 | | |  | | | Telehealth in rehabilitation psychology and neuropsychology | | M. Wells et al. | 2021 |
|  | 83 | | |  | | | The Making and Evaluation of Digital Games Used for the Assessment of Attention: Systematic Review | | K. Wiley et al. | 2021 |
|  | 84 | | |  | | | Tele-neuropsychological assessment tools in Italy: a systematic review on psychometric properties and usability | | E. Zanin et al. | 2021 |
|  | 85 | | |  | | | Rethinking autism spectrum disorder assessment for children during COVID-19 and beyond | | L. Zwaigenbaum et al. | 2021 |
|  | 86 | | |  | | | Internet-based psychotherapies | | G. Andersson | 2022 |
|  | 87 | | |  | | | Characterizing available tools for synchronous virtual assessment of toddlers with suspected autism spectrum disorder: A brief report | | N. Berger et al. | 2022 |
|  | 88 | | |  | | | Integrating telehealth into neurodevelopmental assessment: a model from the Cardiac Neurodevelopmental Outcome Collaborative | | S. Cox et al. | 2022 |
|  | 89 | | |  | | | Best Practice During Teleconsultations With Adolescents: A Scoping Review | | S. Davidson et al. | 2022 |
|  | 90 | | |  | | | Telehealth Interventions to Promote Health and Behavior-Related Outcomes in Adolescents with Autism Spectrum Disorder | | L. Lamash et al. | 2022 |
|  | 91 | | |  | | | Obsessive compulsive disorder during the COVID-19 pandemic: A brief review of course, psychological assessment and treatment considerations | | C. Maye et al. | 2022 |
|  | 92 | | |  | | | A Review on Serious Games for Exercise Rehabilitation | | H. Ning et al. | 2022 |
|  | 93 | | |  | | | Can You See My Screen? Virtual Assessment in Speech and Language | | E. Peña and R. Sutherland | 2022 |
|  | 94 | | |  | | | Review of Innovative Immersive Technologies for Healthcare Applications | | Z. Qu et al. | 2022 |
|  | 95 | | |  | | | What works for whom with telemental health? A rapid realist review | | M. Schlief et al. | 2022 |
|  | 96 | | |  | | | Assessment for an Unprecedented Education | | T. Victorița | 2022 |
|  | ***Self-Assessment/self-help*** | | | | | | | | | |
|  | 1 | | |  | | | Web-based self-help for problem drinkers: A pragmatic randomized trial | | H. Riper et al. | 2008 |
|  | 2 | | |  | | | Transdiagnostic, affect-focused, psychodynamic, guided self-help for depression and anxiety through the internet: study protocol for a randomised controlled trial | | R. Johansson et al. | 2012 |
|  | 3 | | |  | | | Internet and patient empowerment in individuals with symptoms of an eating disorder: A cross-sectional investigation of a pro-recovery focused e-community | | J. J. Aardoom et al. | 2014 |
|  | 4 | | |  | | | Examining the effectiveness and feasibility of a self-guided version of positive affect treatment | | A. G. Loerinc | 2019 |
|  | 5 | | |  | | | Using internet-based self-help to bridge waiting time for face-to-face outpatient treatment for Bulimia Nervosa, Binge Eating Disorder and related disorders: Study protocol of a randomized controlled trial | | B. Vollert et al. | 2019 |
|  | 6 | | |  | | | The Sextherapylondon interactive website for sexual difficulties: Content, design and rationale | | K. Gurney et al. | 2020 |
|  | 7 | | |  | | | Leveraging technology to improve military mental health: Novel uses of smartphone apps | | E. Vermetten et al. | 2020 |
|  | 8 | | |  | | | Feasibility and initial validation of 'HD-Mobile', a smartphone application for remote self-administration of performance-based cognitive measures in Huntington's … | | B. McLaren et al. | 2021 |
|  | 9 | | |  | | | A virtual reality-based self-help intervention for dealing with the psychological distress associated with the COVID-19 lockdown: an effectiveness study with a two … | | G. Riva et al. | 2021 |
|  | 10 | | |  | | | Effects of word familiarity and receptive vocabulary size on speech-in-noise recognition among young adults with normal hearing | | M. Braza et al. | 2022 |
|  | 11 | | |  | | | The association between home stay and symptom severity in major depressive disorder: preliminary findings from a multicenter observational study using … | | P. Laiou et al. | 2022 |
|  | 12 | | |  | | | Use of a Mobile Health (mHealth) Platform for Remote Assessment of Suicidal Ideation, Depression, and Anxiety: A Longitudinal Retrospective Study | | A. Pardes et al. | 2022 |
